# Supplementary material for: Trends in the costs of drugs launched in the UK between 1981 and 2015: an analysis of the launch price of drugs in five disease areas
Source: BMJ Open. 2019 May 5;9(5):e027625. doi: 10.1136/bmjopen-2018-027625 (PMC6501986; doi:10.1136/bmjopen-2018-027625)
Supplement: Supplementary file 1 [file bmjopen-2018-027625supp001.pdf]

Disease area	Drug	Class/Mechanism	Year of launch	Adjusted 28-day launch price (£)	First of kind	Biologic drug	New drug or new indication
Hypertension	Captopril	ACE inhibitor	1981	59.06	Yes	No	New drug
Hypertension	Indoramin	α1-adrenergic receptor antagonist	1981	39.37	No	No	New drug
Hypertension	Dilitiazem	Calcium channel blocker	1984	49.82	No	No	New drug
Hypertension	Enalapril	ACE inhibitor	1985	41.85	No	No	New drug
Hypertension	Nicardipine	Calcium channel blocker	1986	45.52	No	No	New drug
Hypertension	Terazosin	α1-adrenergic receptor antagonist	1987	38.01	No	No	New drug
Hypertension	Bisoprolol fumarate	β-adrenergic receptor antagonist	1988	19.29	No	No	New drug
Hypertension	Lisinopril	ACE inhibitor	1988	47.91	No	No	New drug
Hypertension	Doxazosin	α1-adrenergic receptor antagonist	1989	32.02	No	No	New drug
Hypertension	Isradipine	Calcium channel blocker	1989	41.86	No	No	New drug
Hypertension	Quinapril	ACE inhibitor	1989	49.27	No	No	New drug
Hypertension	Amlodipine	Calcium channel blocker	1990	22.05	No	No	New drug
Hypertension	Perindopril	ACE inhibitor	1990	22.57	No	No	New drug
Hypertension	Ramipril	ACE inhibitor	1990	24.45	No	No	New drug
Hypertension	Celiprolol	β-adrenergic receptor antagonist	1991	17.13	No	No	New drug
Hypertension	Cilazapril	ACE inhibitor	1991	37.74	No	No	New drug
Hypertension	Felodipine	Calcium channel blocker	1991	14.20	No	No	New drug
Hypertension	Fosinopril	ACE inhibitor	1991	36.70	No	No	New drug
Hypertension	Esmolol	β-adrenergic receptor antagonist	1992	9.93	No	No	New drug
Hypertension	Lacidipine	Calcium channel blocker	1992	23.73	No	No	New drug
Hypertension	Trandolapril	ACE inhibitor	1992	20.86	No	No	New drug
Hypertension	Carvedilol	β-adrenergic receptor antagonist	1994	19.28	No	No	New drug
Hypertension	Torasemide	Loop diuretic	1994	7.41	No	No	New drug
Hypertension	Losartan	Angiotensin-II receptor antagonist	1995	27.26	Yes	No	New drug
Hypertension	Moxonidine	Imidazoline I_1 receptor agonist	1996	15.82	Yes	No	New drug
Hypertension	Valsartan	Angiotensin-II receptor antagonist	1996	23.85	No	No	New drug
Hypertension	Candesartan	Angiotensin-II receptor antagonist	1997	23.24	No	No	New drug
Hypertension	Irbesartan	Angiotensin-II receptor antagonist	1997	25.41	No	No	New drug
Hypertension	Lercanidipine	Calcium channel blocker	1998	14.77	No	No	New drug
Hypertension	Imidapril	ACE inhibitor	1999	9.51	No	No	New drug
Hypertension	Nebivolol	β-adrenergic receptor antagonist	1999	13.91	No	No	New drug
Hypertension	Eprosartan	Angiotensin-II receptor antagonist	2000	20.82	No	No	New drug
Hypertension	Telmisartan	Angiotensin-II receptor antagonist	2000	17.78	No	No	New drug
Hypertension	Olmesartan	Angiotensin-II receptor antagonist	2003	18.68	No	No	New drug
Hypertension	Aliskiren	Renin inhibitor	2007	23.71	Yes	No	New drug
Hypertension	Azilsartan medoxomil	Angiotensin-II receptor antagonist	2012	17.73	No	No	New drug
Asthma	Reproterol hydrochloride aerosol inhalation	Selective β2-agonist (short acting)	1981	9.22	No	No	New drug
Asthma	Reproterol hydrochloride tablets	Selective β2-agonist (short acting)	1981	14.20	No	No	New drug
Asthma	Hydrocortisone acetate	Corticosteroid	1982	8.64	No	No	New indication
Asthma	Budesonide	Corticosteroid	1983	14.59	No	No	New drug
Asthma	Pirbuterol aerosol inhalation	Selective β2-agonist (short acting)	1983	10.93	No	No	New drug
Asthma	Nedocromil sodium	Cromoglicate	1987	42.74	No	No	New drug
Asthma	Salmeterol	Selective β2-agonist (long acting)	1991	49.22	Yes	No	New drug
Asthma	Tulobuterol hydrochloride	Selective β2-agonist (long acting)	1991	22.37	No	No	New drug

Asthma	Bambuterol hydrochloride	Selective β2-agonist (long acting)	1993	20.27	No	No	New drug
Asthma	Pirbuterol tablets	Selective β2-agonist (short acting)	1993	12.13	No	No	New drug
Asthma	Fluticasone propionate	Corticosteroid	1994	17.53	No	No	New drug
Asthma	Formoterol fumarate	Selective β2-agonist (long acting)	1996	37.77	No	No	New drug
Asthma	Montelukast	Leukotriene receptor antagonists	1998	37.97	Yes	No	New drug
Asthma	Zafirlukast	Leukotriene receptor antagonists	1999	37.19	No	No	New drug
Asthma	Mometasone furoate	Corticosteroid	2003	29.74	No	No	New indication
Asthma	Ciclesonide	Corticosteroid	2006	9.67	No	No	New drug
Asthma	Omalizumab	Anti IgE humanised monoclonal antibody	2006	764.45	Yes	Yes	New drug
Asthma	Fluticasone furoate and vilanterol	Selective β2-agonist (long acting)	2014	26.43	No	No	New drug
Schizophrenia	Levomepromazine maleate	First generation antipsychotic	1983	20.22	No	No	New drug
Schizophrenia	Zuclopenthixol dihydrochloride	First generation antipsychotic	1983	19.41	No	No	New drug
Schizophrenia	Sulpiride #	Atypical antipsychotic	1984	139.22	No	No	New drug
Schizophrenia	Clozapine	Atypical antipsychotic	1990	331.80	Yes	No	New drug
Schizophrenia	Loxapine	First generation antipsychotic	1990	31.75	No	No	New drug
Schizophrenia	Remoxipride	Atypical antipsychotic	1991	39.75	No	No	New drug
Schizophrenia	Risperidone	Atypical antipsychotic	1993	189.15	No	No	New drug
Schizophrenia	Olanzapine	Atypical antipsychotic	1997	200.04	No	No	New drug
Schizophrenia	Sertindole	Atypical antipsychotic	1997	155.55	No	No	New drug
Schizophrenia	Amisulpride	Atypical antipsychotic	1998	93.95	No	No	New drug
Schizophrenia	Quetiapine	Atypical antipsychotic	1998	214.80	No	No	New drug
Schizophrenia	Zotepine	Atypical antipsychotic	1999	182.40	No	No	New drug
Schizophrenia	Aripiprazole	Atypical antipsychotic	2004	131.25	No	No	New drug
Schizophrenia	Paliperidone	Atypical antipsychotic	2008	112.92	No	No	New drug
Schizophrenia	Lurasidone	Atypical antipsychotic	2014	92.39	No	No	New drug
Rheumatoid arthritis	Tiaprofenic acid	Non-steroidal anti-inflammatory drug (NSAID)	1982	58.61	No	No	New drug
Rheumatoid arthritis	Etodolac	Non-steroidal anti-inflammatory drug (NSAID)	1985	71.92	No	No	New drug
Rheumatoid arthritis	Sulfasalazine	Aminosalicylate	1987	23.59	Yes	No	New indication
Rheumatoid arthritis	Nabumetone	Non-steroidal anti-inflammatory drug (NSAID)	1987	40.52	No	No	New drug
Rheumatoid arthritis	Tenoxicam	Non-steroidal anti-inflammatory drug (NSAID)	1988	40.38	No	No	New drug
Rheumatoid arthritis	Acemetacin	Non-steroidal anti-inflammatory drug (NSAID)	1991	28.64	No	No	New drug
Rheumatoid arthritis	Methotrexate	Dihydrofolate reductase inhibitor	1992	2.22	Yes	No	New indication
Rheumatoid arthritis	Ciclosporin	Calcineurin inhibitor	1994	240.63	Yes	No	New indication
Rheumatoid arthritis	Aceclofenac	Non-steroidal anti-inflammatory drug (NSAID)	1996	23.45	No	No	New drug
Rheumatoid arthritis	Meloxicam	Non-steroidal anti-inflammatory drug (NSAID)	1996	21.81	No	No	New drug
Rheumatoid arthritis	Leflunomide	Pyrimidine synthesis inhibitor	1999	69.71	Yes	No	New drug
Rheumatoid arthritis	Infliximab	TNF-α inhibitor	1999	1052.22	Yes	Yes	New indication
Rheumatoid arthritis	Rofecoxib	Selective cyclo-oxygenase 2 (COX-2) inhibitor	1999	30.12	Yes	No	New drug
Rheumatoid arthritis	Celecoxib	Selective cyclo-oxygenase 2 (COX-2) inhibitor	2000	25.97	No	No	New drug
Rheumatoid arthritis	Etanercept	TNF-α inhibitor	2000	920.27	No	Yes	New drug
Rheumatoid arthritis	Anakinra	IL-1 inhibitor	2002	888.47	Yes	Yes	New drug
Rheumatoid arthritis	Etoricoxib	Selective cyclo-oxygenase 2 (COX-2) inhibitor	2002	33.80	No	No	New drug
Rheumatoid arthritis	Adalimumab	TNF-α inhibitor	2003	946.83	No	Yes	New drug
Rheumatoid arthritis	Valdecoxib	Selective cyclo-oxygenase 2 (COX-2) inhibitor	2003	28.12	No	No	New drug
Rheumatoid arthritis	Rituximab	CD20 inhibitor	2006	330.65	Yes	Yes	New indication
Rheumatoid arthritis	Abatacept	Inhibitor of T-cell co-stimulation	2007	900.14	Yes	Yes	New drug

Rheumatoid arthritis	Tocilizumab	IL-6 inhibitor	2009	980.44	Yes	Yes	New drug
Rheumatoid arthritis	Certolizumab pegol	TNF-α inhibitor	2009	781.10	No	Yes	New drug
Rheumatoid arthritis	Golimumab	TNF-α inhibitor	2010	792.23	No	Yes	New drug
Colorectal cancer	Folinic acid + Fluorouracil (5FU)	Thymidylate synthase inhibitor	1992	83.04	No	No	New indication
Colorectal cancer	Raltitrexed	Thymidylate synthase inhibitor	1996	588.05	No	No	New drug
Colorectal cancer	Irinotecan	DNA topoisomerase I inhibitor	1997	1452.13	Yes	No	New drug
Colorectal cancer	Oxaliplatin	Platinum-based chemotherapeutic drug	2000	1195.80	Yes	No	New drug
Colorectal cancer	Capecitabine	Thymidylate synthase inhibitor	2001	507.49	No	No	New drug
Colorectal cancer	Tegafur (with uracil)	Thymidylate synthase inhibitor	2001	876.52	No	No	New drug
Colorectal cancer	Cetuximab	EGFR inhibitor	2005	1105.20	Yes	Yes	New drug
Colorectal cancer	Bevacizumab	VEGF inhibitor	2006	4200.67	Yes	Yes	New drug
Colorectal cancer	Panitumumab	EGFR inhibitor	2008	2981.95	No	Yes	New drug
Colorectal cancer	Regorafenib	Multi-tyrosine kinase inhibitor	2013	3888.39	Yes	No	New drug
Colorectal cancer	Aflibercept	VEGF inhibitor	2013	614.11	No	Yes	New drug

Clozapine is usually considered the first atypical antipsychotic to be launched, sulpiride was reclassified later.
