# Supplementary material for: Trends in the costs of drugs launched in the UK between 1981 and 2015: an analysis of the launch price of drugs in five disease areas
Source: BMJ Open. 2019 May 5;9(5):e027625. doi: 10.1136/bmjopen-2018-027625 (PMC6501986; doi:10.1136/bmjopen-2018-027625)
Supplement: Author's manuscript [file bmjopen-2018-027625.draft_revisions.pdf]

BMJ Open is committed to open peer review. As part of this commitment we make the peer review history of every article we publish publicly available.

When an article is published we post the peer reviewers' comments and the authors' responses online. We also post the versions of the paper that were used during peer review. These are the versions that the peer review comments apply to.

The versions of the paper that follow are the versions that were submitted during the peer review process. They are not the versions of record or the final published versions. They should not be cited or distributed as the published version of this manuscript.

BMJ Open is an open access journal and the full, final, typeset and author-corrected version of record of the manuscript is available on our site with no access controls, subscription charges or pay-per-view fees (http://bmjopen.bmj.com).

If you have any questions on BMJ Open's open peer review process please email info.bmjopen@bmj.com

BMJ Open

Trends in the costs of new drugs launched in the United Kingdom between 1981 and 2015

Journal:	*BMJ Open*
Manuscript ID	bmjopen-2018-027625
Article Type:	Research
Date Submitted by the Author:	31-Oct-2018

Complete List of Authors:	Ward, Derek; University of Birmingham, Institute of Applied Health Reseach<br>Doos, Lucy; University of Birmingham, College of Medical and Dental Sciences<br>Stevens, Andrew; University of Birmingham, College of Medical and Dental Sciences

Keywords:	Drug pricing, Trends, pharmaceutical innovation, United KIngdom

SCHOLARONE™
Manuscripts

Trends in the costs of new drugs launched in the United Kingdom between 1981 and 2015

Derek J Ward[1], Lucy Doos[2], Andrew Stevens[1].

1. Institute of Applied Health Research
   University of Birmingham, Birmingham, United Kingdom
2. College of Medical and Dental Sciences
   University of Birmingham, Birmingham, United Kingdom

Corresponding author:

Dr Derek Ward d.j.ward@bham.ac.uk Tel. 0121 414 7660

Murray Learning Centre, Institute of Applied Health Research, University of Birmingham, Edgbaston, Birmingham, B15 2TT, United Kingdom

Word count (excluding title page, abstract, references, figures and tables): 3,231

Keywords: drug pricing, trends, pharmaceutical innovation, United Kingdom

For peer review only

ABSTRACT

Objectives: To investigate the trend in launch price of new drugs for five common health conditions.

Design: Cross-sectional study using data on new drugs launched in the UK between 1981 and 2015 for hypertension, asthma, rheumatoid arthritis, schizophrenia and colorectal cancer.

Data and sources: All drugs marketed in the UK between 1981 and 2015 (inclusive), and licensed specifically for the treatment of one of the five chosen conditions were included in the study. Newly launched medicines and their launch prices were identified by hand-searching all editions of the British National Formulary (BNF) in addition to searching the websites of relevant regulatory agencies (European Medicines Agency and Medicines and Healthcare products Regulatory Agency). The launch price in UK pounds for a 28-day supply of each medicine at a typical or usual maintenance dose was adjusted for the effects of general inflation using the Gross Domestic Product (GDP) deflator series.

Results: 105 drugs were included in our study with a mean inflation adjusted 28-day launch price of £261 (SD £584). The launch price of new drugs varied significantly across the five conditions, with drugs for hypertension having the lowest mean price (£27) and drugs for colorectal cancer having the highest mean price (£1,245) ($p<0.000$). There were large increases in launch prices across the study period, but the magnitude and pattern was markedly different between therapeutic areas. Biologic drugs represented 13.3% of all included drugs and had a significantly higher launch price than non- biologic drugs (£1233 vs £111, $p<0.000$). 22.9% of included drugs were first of kind and had a significantly higher launch price than follow-on drugs (££630 vs £151) ($p<0.0001$).

For peer review only

Conclusion: Drugs prices continue to increase across different therapeutic areas. This has some association with novelty, but, it is not clear if this increase in price is associated with medical benefits.

For peer review only

For peer review only - http://bmjopen.bmj.com/site/about/guidelines.xhtml

ARTICLE SUMMARY

Strengths and limitations of this study

- The timeline of this study enables a very long-term view of drug pricing that goes beyond previous published work.

- This study used the British National Formulary to identify new drugs and new licensed indications for existing marketed drugs and is therefore likely to represent a comprehensive view of drug pricing in the UK.

- This study is restricted to publicly available pricing data in the UK; the actual price paid by healthcare providers for drugs may vary from this and the results may not be applicable to other settings.

- This study chose to focus on five health conditions in a pragmatic way, and the results may not be generalisable to drugs licensed for use in other health conditions.

For peer review only

BACKGROUND

Over last few decades, spending on healthcare has risen faster than economic growth in many developed countries.[1;2] Internationally, spending on pharmaceuticals represents a significant proportion of the total healthcare budget.[3] For example, high-income countries within the Organisation for Economic Co-operation and Development (OECD) spend on average 18% of their total healthcare expenditure on medicines and this figure can reach up to 80% in some low- and middle-income countries.[3] In the UK, the cost of medicines represented 11.6% of total healthcare expenditure in 2008.[1]

Worldwide, affordability is a major component of ensuring access to essential medicines for many conditions.[4,5] Many publicly funded healthcare providers, including the UK National Health Service (NHS), aim to provide effective treatment at a price that represents valuable for money.[6] Healthcare systems in many countries use a variety of cost-saving and cost-containing measures, including ensuring approval and reimbursement of drugs is dependent on an assessment of clinical and cost effectiveness using Health Technology Assessment (HTA) in order to counter financial challenges.[6] An understanding of the drivers of medicine prices is therefore important particularly where countries and policy makers are seeking to develop pricing policies that improve both the availability and the affordability of such medicines.[3]

The increasing cost of pharmaceuticals used to manage a number of common conditions has received increasing attention in recent years.[4,7] In the USA, retail prescription drug spending accelerated in 2014, growing 13.1% in one year, representing the largest annual increase since 2003. According to a recent report that considered the impact of changes in the pharmaceutical industry and its impact on healthcare payers in the USA, this increase was the result of increasing demand and changes in patient behaviour, which both had a significant impact on drug expenditures.

For peer review only

However, innovation or novelty is also a factor, as pricing of first of kind drugs was noted to be one of the most important factors driving this trend.[8] Studies on rising overall pharmaceutical expenditure seen during the 1990s to mid-2000s in North America and Europe have highlighted the role played by both increased utilisation and the adoption of newer, more expensive medicines.[9,10] The high cost of newer agents has been identified by some commentators as the key component of rising per-patient pharmaceutical costs for cancer chemotherapy and the treatment of diabetes mellitus, glaucoma, psychosis, multiple sclerosis and haemophilia.[11-12] However, other studies suggest increasing utilisation as the major driver of rising costs in the treatment of hypertension, hyperlipidaemia, depression, and rheumatoid arthritis, where increasing overall pharmaceutical spending has not necessarily been accompanied by increased per-patient spending.[13-15]

Increases in the cost of newer medicines has been variously explained as due to increasing regulatory and technology assessment requirements, research and development costs, attrition rates, production costs, the patent system, and marketing practice.[16-22]

To further investigate trends in the price of pharmaceuticals, we sought to describe long-term, 35-year trends in the launch price for all new medicines marketed in the UK to treat one of five common conditions that were chosen to represent a range of physical and mental health disorders for which significant pharmaceutical innovation had occurred during the period of interest.

For peer review only

METHODS

Identification of new drugs

All medicines marketed in the UK for the first time between 1981 and 2015 (inclusive) for the treatment of one the following five conditions were included in the study using International Statistical Classification of Diseases version 10 (ICD-10)[23] : 1) Asthma (ICD-10 code J45), 2) Colorectal cancer (ICD-10 code C18), 3) Essential hypertension (ICD-10 code I10), 4) Rheumatoid arthritis (seropositive rheumatoid arthritis [ICD-10 codes M05 and M06]), and 5) Schizophrenia (ICD-10 code F20).

Our study included medicines specifically licensed for the treatment of one of these conditions at the time of first UK launch (as described in the Summary of Product Characteristics [SmPC] published by the European Medicines Agency [EMA][24] and Medicines and Healthcare products Regulatory Agency [MHRA][25], as appropriate) as well as existing licensed drugs that subsequently received Marketing Authorisation for one of the five study conditions, including those that have subsequently been withdrawn.

Medicines were identified by hand-searching all editions of the British National Formulary (BNF)[26] published between 1981 and 2015. The BNF lists all medicines available for prescription and dispensing within the UK irrespective of whether they are available on the NHS; the date of first appearance in the BNF (for new medicines) or the date where the indication was amended in the BNF (for existing licensed medicines subsequently receiving Marketing Authorisation for one of the specified conditions) was taken to be the year of launch in the UK. Medicines listed in BNF edition 1 were considered to already be available for use in 1981, and therefore excluded from the study. This approach to identifying all relevant new medicines was supplemented by searches of the electronic Medicines Compendium (eMC [Datapharm Communications Ltd.])[27], commercial pharmaceutical databases (Adis Insight [Springer][28] and Pharmaprojects [Informa Healthcare])[29], the websites of

For peer review only - http://bmjopen.bmj.com/site/about/guidelines.xhtml

relevant regulatory agencies (EMA and MHRA)[24,25], and searches for clinical practice guidelines issued by the National Institute for Health and Clinical Excellence (NICE)[30] and relevant specialist clinical societies and associations.

Price calculation

The launch price in UK pounds for a 28-day supply of each medicine at a typical or usual maintenance dose (including cycles or courses where relevant) was calculated from the unit price provided in the edition of the BNF in which the medicine first appeared. Initial loading or lower introductory doses were ignored. The published SmPC and/or relevant clinical guidelines were consulted where no usual dose was listed in the BNF. The dose range mid-point was used where no usual dose could be established (adjusted to be achievable using available dose formulations). In all cases, the price was calculated using the least expensive combination of available dose formulations (where relevant, this assumed wastage, a body weight of 76.9kg, and body surface area of 1.7m^2).

The 28-day launch prices for all medicines were adjusted for the effects of general inflation using the Gross Domestic Product (GDP) deflator series.[31] GDP deflators serve as a measure of inflation within the economy as a whole for a given time period and were used to adjust prices to 2015 values, thereby allowing direct comparisons between prices at different time points.

We excluded new combinations of existing licensed medicines and those not found in the BNF.

Data handling and analysis

Data on drug name, whether it was newly launched or an existing licensed and available product, the mechanism of action or drug class, whether the drug was a biological agent, launch year, launch price for a typical 28-day supply and 2015 adjusted 28-day launch price were extracted and entered onto an Excel spreadsheet. Statistical analysis was carried out using IBM SPSS (version 21) for

For peer review only - http://bmjopen.bmj.com/site/about/guidelines.xhtml

Windows. Trends in adjusted 28-day launch prices were initially explored using simple descriptive statistics and scatter plots. Descriptive analyses were presented as means and standard deviations (SD) for normally distributed continuous variables. Significant differences were determined using ANOVA for continuous normally distributed data and X^2 for dichotomous variables.

For peer review only

RESULTS

105 drugs met our inclusion criteria, and were launched with an inflation-adjusted 28-day price that ranged from £2.20 (methotrexate for rheumatoid arthritis, 1992) to £4,200.70 (bevacizumab for colorectal cancer, 2006) (Table 1). Nearly one third of the drugs were launched for hypertension (34.3%), while just 11.4% were launched for colorectal cancer. There were statistically significant differences in the mean inflation-adjusted 28-day launch price by condition, with drugs for hypertension having the lowest mean price (£27) and drugs for colorectal cancer having the highest mean price (£1,245) (p<0.0001).

Drug characteristics

The majority of the drugs included in the study were newly launched (91.4%) as opposed to new licensed indications for existing marketed drugs (8.9%). The mean inflation-adjusted 28-day price for newly launched drugs was higher than that for new licensed indications for existing marketed drugs. This pattern was observed across all three relevant conditions (asthma, rheumatoid arthritis and colorectal cancer), but the differences were not statistically significant (Table 2).

Fourteen (13.3%) of the included drugs were biologic agents, one third of which were for colorectal cancer and none of which were for hypertension or schizophrenia. Over the three relevant conditions, biologic drugs had a significantly higher mean inflation-adjusted 28-day launch price than non-biologic drugs (£1,233 vs £111, p<0.000).

Newly launched biologics had nearly twice the mean inflation-adjusted 28-day launch price of newly licensed indications for existing marketed biologic drugs (£1322 vs £691), however this difference was not statistically significant.

For peer review only - http://bmjopen.bmj.com/site/about/guidelines.xhtml

Table 1: Number of drugs and their 28-day inflation-adjusted launch price by selected condition launched in the period 1981 to 2015 (inclusive)

Includes newly licensed drugs as well as new licensed indications for existing marketed drugs. Prices adjusted to 2015 values using the Gross Domestic Product (GDP) deflator series.

**Selected condition**	**Number (%)**	**Mean price (SD)**	**Median price (range)**
Hypertension	36 (34.3%)	£27.01 (13.09)	£51.70 (7.40-59.1)
Asthma	18 (17.1%)	£64.73 (175.10)	£755.80 (8.60-764.50)
Schizophrenia	15 (14.3%)	£130.31 (86.56)	£312.40 (19.40-331.80)
Rheumatoid arthritis	24 (22.9%)	£347.16 (414.24)	£1050.00 (2.20-1052.20)
Colorectal cancer	12 (11.4%)	£1245.45 (1210.62)	£4156.40 (44.30-4200.70)
Total	105	£260.66 (583.66)	£4198.50 (2.20-4200.70)

For peer review only

Table 2: Mean 28-day inflation-adjusted launch price by selected condition according to biologic vs non-biologic drug type, first of kind vs follow on drug, and new drug vs new indication for existed marketed drug, 1981-2015.

Selected condition	Biologic drug	Non-biologic drug	First of kind	Follow on	New drug	New licensed indication
Hypertension			£31.50	£26.50		
			P=0.47			
Asthma	£764.50	£23.60	£283.90	£20.90	£70.40	£19.20
	p<0.001		p=0.01		p=0.71	
Schizophrenia			£331.80	£115.90		
			p=0.01			
Rheumatoid arthritis	£813.90	£211.90	£451.80	£227.40	£389.70	£329.90
	p=0.001		p=0.31		p=0.79	
Colorectal cancer	£2762.60	£739.70	£1549.00	£941.80	£1481.80	£63.70
	p=0.004		p=0.41		p=0.14	
All conditions	£1232.90	£111.10	£630.10	£151.20	£266.20	£201.70
	p<0.001		p<0.001		p=0.75	

For peer review only

Pattern of change in pricing

A large increase in inflation-adjusted 28-day launch prices was observed when all drugs included in this study were considered together. However, the magnitude and pattern of change varied considerably between the five therapeutic areas (Figure 1).

For hypertension, following the introduction of captopril in 1981, the typical price of newly launched drugs generally fell (with some variation) until the early 1990s, after which prices appeared to stabilise. For asthma, there was a single high-cost outlier (omalizumab, launched in 2006), but there was also a sharp increase in launch price in 1987 with the launch of nedocromil sodium (a cromoglicate), which was nearly three times the inflation-adjusted price of the previously launched drug. The overall trend in the price of newly launched drugs for schizophrenia was broadly flat. However, there were some significant variations across the period of interest. In particular, two drugs launched in 1990 showed a great discrepancy in inflation-adjusted 28-day launch price, with the price of clozapine (£332.80, the first atypical antipsychotic launched) being nearly ten-fold higher than loxapine £31.75), which was launched the same year. For rheumatoid arthritis launch prices experienced a marked upward step in 1999 with the arrival of infliximab (TNF-α inhibitor), the first biologic agent licensed for this condition. This was followed by a slight decrease in the inflation-adjusted price the following year when the next drug was launched (by 12.5%). Launch prices for drugs licensed to treat colorectal cancer rose increasingly rapidly in the two decades following the mid-1990s. In particular, the introduction of bevacizumab in 2006, the first biological drug for this condition, resulted in a nearly threefold increase in the inflation-adjusted 28-day launch price compared to drugs launched in the previous few years. However, in more recent years, the pattern has changed to one of falling prices with the introduction of newer biologic drugs and the non-biologic drug regorafenib (in 2013).

For peer review only - http://bmjopen.bmj.com/site/about/guidelines.xhtml

Drug class/mechanism of action

The 105 drugs included in our study represented 34 different drug classes or mechanisms of action, 17 of which were represented only once. There were significant variations in the inflation-adjusted 28-day launch price for drugs by drug class/mechanism of action; these differences were statistically significant across all but one of the therapeutic areas (the exception being drugs for colorectal cancer) (Appendix1).

Among the 36 drugs from nine drug classes included for hypertension, α1-adrenergic receptor antagonists had the highest mean inflation-adjusted 28-day launch price (£36.50) followed by angiotensin converting enzyme (ACE) inhibitors (£35.00). For asthma, the anti IgE humanised monoclonal antibody omalizumab had the highest inflation-adjusted launch price by far (£764.45). The next most expensive was the single cromoglicate (£42.74), followed by leukotriene receptor antagonists (£37.58) and selective β2-agonists – long acting (£31.21). The lowest mean inflation-adjusted 28-day launch price was for selective β2-agonists – short acting (£11.62). For rheumatoid arthritis, nearly one third of drugs (29.2%) were non-steroidal anti-inflammatory drugs (NSAIDs), and there was a marked downward trend in their launch prices between 1985 and 1996 (mean adjusted 28-day launch price for the whole period £40.76). TNF-α inhibitors were the second common group of drugs launched for rheumatoid arthritis, and these had a mean inflation-adjusted launch price of £898.53, though single examples of an IL-1 inhibitor, an inhibitor of T-cell co-stimulation and IL-6 inhibitors were priced even higher. Almost half of all new drugs launched for colorectal cancer were thymidylate synthase inhibitors, which had a mean inflation-adjusted 28-day launch price £419.87. The launch price for this class of drugs showed a dramatic 1,880% increase between 1985 and 2001, but even so the mean launch price was still the lowest of all drug classes/mechanisms of action identified for this condition. Drugs for schizophrenia were grouped into two broad categories, recognising the difficulty of ascribing a specific class or individual mechanism of action. Atypical

For peer review only

antipsychotics had significantly higher inflation-adjusted 28-day launch price (£156.93) than first generation antipsychotics (£23.80) ($p<0.01$).

First of kind and follow-on drugs

Less than a quarter of the drugs included in our study (22.9%) represented the first of a new class of drugs or a new mechanism of action (first of kind). However, these drugs had a significantly higher inflation-adjusted 28-day launch price than follow-on drugs (£630 vs £151, $p<0.0001$) (Table 2). However, within therapy areas a statistically significant difference between first of kind and follow-on drugs was only observed for asthma and schizophrenia (both $p<0.01$). First of kind drugs represented half of all newly launched drugs for colorectal cancer, a proportion that was greater than for any other indication.

We identified ten examples where the first of kind and at least one follow-on drug were launched in the time period of this study; three of these drug classes/mechanisms of action were licensed for colorectal cancer, two each for asthma, hypertension and rheumatoid arthritis, and one for schizophrenia. Excluding drugs for colorectal cancer, there was only one instance in which the inflation-adjusted launch price for a follow-on drug was greater than that for the first of kind (the exception was etoricoxib for rheumatoid arthritis, where the launch price was 12% greater than the first of kind). In colorectal cancer, all new thymidylate synthase inhibitors were priced above the first of kind (fluorouracil), and the EGFR inhibitor panitumumab was priced over 160% higher than cetuximab, the first of kind. Where more than one follow-on drug was included in our dataset, it was apparent that inflation-adjusted launch prices generally fell over time, particularly as the time from when the first of kind was launched increased (Figure 2). The overall decrease in inflation-adjusted 28-day launch price from the first of kind to the final launch of a follow on varied between -£2.00 (selective cyclo-oxygenase 2 inhibitors for rheumatoid arthritis) to -£259.99 (TNF-α inhibitors for rheumatoid arthritis), and in relative terms varied between -6.6% (selective cyclo-oxygenase 2

For peer review only - http://bmjopen.bmj.com/site/about/guidelines.xhtml

inhibitors for rheumatoid arthritis) to -83.9% (ACE inhibitors for hypertension). The exception was the thymidylate synthase inhibitors for colorectal cancer, the launch prices for which increased dramatically during the study period (by £832.25 or 1880%).

For peer review only

DISCUSSION

Main findings

Our findings show that while the launch prices of drugs have generally increased over the study period, after controlling for inflation there were marked differences according to therapeutic area, with some showing no real-terms increase. In addition, the launch price of first of kind drugs is consistently higher than that of other drugs, and that biological agents are considerably more expensive at launch that other drugs marketed for the same condition.

Pharmaceutical companies argue that the price of new drugs is set at a level needed to recoup the vast costs incurred in bringing that drug to market, as well as fund future research and development.[32-34] These costs have risen over time for a number of reasons, including the increasing complexity and length of clinical trial programmes to meet the needs of regulators and HTA bodies,[35-38] so that by some estimates, the rate of new drug launches per R&D spend has fallen by around a half every nine years since the 1950s.[39] However, R&D costs per drug candidate are not transparent for commercial reasons, and the oft quoted cost of bringing a new drug to market may fail to properly account for publicly funded basic and translational research.[33] General pressures on pharmaceutical developers' R&D costs do not explain the marked variations in the launch price of drugs across the five therapeutic areas that we observed in our study. In particular, the mean inflation-adjusted 28-day launch price for drugs indicated for colorectal cancer was 46 times greater than the price of those for hypertension. This finding agrees with the general observation made by commentators that managing cancer is expensive and the prices for drugs associated with cancer represent a great burden to healthcare systems. [35,40] Howard et al[35] reported that the average adjusted launch price of anticancer drugs increased by 10% annually between 1995 and 2013, which is considerably less than the increase in inflation-adjusted launch price of drugs for colorectal cancer we observed in the period 1985 to 2013. Launch prices spiked in 2006 with the introduction of the first biological drug, bevacizumab, which might be expected given the increased development and

For peer review only - http://bmjopen.bmj.com/site/about/guidelines.xhtml

manufacturing costs associated with biological agents.[33] This pattern of increased drug costs associated with the introduction of the first biological drug mirrored that seen in rheumatoid arthritis, but unlike rheumatoid arthritis, commentators have argued that this pressure on prices seen in cancer is not related to the magnitude of expected health benefit.[35]

We also found that first of kind drugs were more expensive at launch and that where our data included both first of kind and follow on drugs, the price of subsequent launches was lower but still rather closely related to that of the first launch. This pattern is readily explained by the market into which new and follow on drugs are launched, with first of kind drugs competing with established drugs for the same condition or creating a new market if no equivalent treatment exists. The premium given to first of kind drugs could be justified if payers are willing to pay more to receive a substantial therapeutic advance compared to existing drug or other treatment options.[31] In our data, this may partly explain the very marked differences between areas where there is limited scope for new drugs to markedly improve care. The idea of 'rewardable innovation' suggests that health services and patients place value on improvements in a wide range of domains, including those related to new molecular structures, drug synthesis, pharmacodynamics, pharmacokinetics, drug delivery, pharmacogenetics and application in clinical practise.[41] However, many newly launched drugs do not offer any therapeutic advance for patients[40] and even many first of kind drugs do not provide any clinically significant innovation.[42]

Strengths and limitations of the study

We believe our study includes all drugs newly launched in the UK for the five indicated conditions over a 35 year period. However, we chose the five conditions on a pragmatic basis, and generalisability from our findings cannot be assumed, particularly in drugs for rare or orphan conditions, which were not part of our study. In addition, the published list price for drugs may not be actual price paid by NHS organisations, particularly for the more recently launched drugs, as

For peer review only - http://bmjopen.bmj.com/site/about/guidelines.xhtml

patient access schemes have become more common, and we cannot directly account for different drug development costs across different disease areas.

Conclusions

While prices for new drugs continue to increase overall, this masks significant disparities between therapeutic areas. It is unclear to what extent these patterns represent developers passing on real differences in development and manufacturing costs, or different pricing strategies based on an assessment of the current market and payers willingness to pay more for some conditions. Increased prices could be justified where new drugs meet an unmet clinical need [41,42] and could encourage developers to focus efforts in priority fields, where higher risk might reasonably be rewarded by higher returns. Publicly funded healthcare systems demand value for money, but existing pricing systems lack the transparency to ensure access to drugs at reasonable and justified prices.

For peer review only

For peer review only - http://bmjopen.bmj.com/site/about/guidelines.xhtml

CONTRIBUTION OF THE AUTHORS

DJW and AJS conceived the original study idea, and LD contributed to the development of the study design and methods. LD extracted the data, which was independently reviewed by DW. LD and DW conducted the analyses. All authors were involved in the interpretation of the results. LD produced the initial draft of the paper, which was then circulated repeatedly to all authors for critical revision. LD, DJW and AJS read and approved the final version. All authors had full access to all of the study data (including statistical reports and tables), and can take responsibility for the integrity of the data and the accuracy of the analysis. DJW is the guarantor.

COMPETING INTERESTS

All authors have completed the ICMJE uniform disclosure form at www.icmje.org/coi_disclosure.pdf and declare: all authors have financial support from the National Institute for Health Research (NIHR) for the submitted work; all authors have no financial relationships with any organisations that might have an interest in the submitted work in the previous three years; and all authors have no other relationships or activities that could appear to have influenced the submitted work.

FUNDING STATEMENT

The study was undertaken as part of the research programme of the NIHR Horizon Scanning Research & Intelligence Centre (NIHR HSRIC). HSRIC is funded by the National Institute for Health Research (NIHR). The NIHR had no role in the study design; in the collection, analysis and interpretation of the data; in the preparation of the manuscript; or in the decision to submit the article for publication. This article presents independent research funded by the NIHR. The views expressed are those of the authors and not necessarily those of the NHS, the NIHR or the Department of Health.

For peer review only - http://bmjopen.bmj.com/site/about/guidelines.xhtml

DATA SHARING STATEMENT

The dataset used for analyses has been provided as a supplementary file, and will also be added to the Dryad repository, which will provide a permanent, citable and open access home for the dataset. No additional unpublished data are available.

For peer review only

For peer review only - http://bmjopen.bmj.com/site/about/guidelines.xhtml

REFERENCES

1. Organisation for Economic Co-operation and Development. OECD health data 2011. 30 June 2011. Paris, France, OECD.

2. Squires DA, The Commonwealth Fund. Explaining the high health care spending in the United States: an international comparison of supply, utilization, prices, and quality. 1595 Vol. 10. 2012. The Commonwealth Fund. Issues in International Health Policy.

3. Ud-Din Babar Z, Lessing C, Mace C, Bissell K. The Availability, pricing and affordability of three essential asthma medicines in 52 low- and middle-income countries. PharmacoEconomics 2013 31:1063–1082.

4. Creese A, Gasman N, Mariko M. The World Medicines Situation. Geneva: World Health Organization; 2004. http://apps.who.int/medicinedocs/en/d/Js6160e/ Accessed 19 July 2016.

5. Vogler S, Martikainen JE. Pharmaceutical pricing in Europe. In: Zaheer-Ud-Din Babar (ed): Pharmaceutical Prices in the 21st Century: Springer International Publishing Switzerland 2015.

6. Morrison EE, Webb DJ. UK Health Technology Assessment and value based pricing. In: Zaheer-Ud-Din Babar (ed): Pharmaceutical Prices in the 21st Century: Springer International Publishing Switzerland 2015.

7. Cohn J. The Milbank Quarterly 2016; 94(2):260-263.

8. The 2014 drug trend report. Workplace health and benefit. Medical Benefits 30 April 2015; 32 (8): 1-3.

9. Morgan S. Drug spending in Canada: recent trends and causes. Medical Care 2004; 42(7):635-642.

10. Morgan SG. Quantifying components of drug expenditure inflation: the British Columbia seniors' drug benefit plan. Health Services Research 2002; 37(5):1243-1266.

For peer review only - http://bmjopen.bmj.com/site/about/guidelines.xhtml

11. Halbert RJ, Zaher C, Wade S, Malin J, Lawless GD, Dubois RW. Outpatient cancer drug costs: changes, drivers, and the future. Cancer 2002; 94(4):1142-1150.

12. Schrag D. The price tag on progress--chemotherapy for colorectal cancer. New England Journal of Medicine 2004; 351(4):317-319.

13. Koskinen H, Martikainen JE, Maljanen T. Antipsychotics and antidepressants: an analysis of cost growth in Finland from 1999 to 2005. Clinical Therapeutics 2009; 31(pt 1):1469-77.

14. Chen Y, Kelton CM, Jing Y, Guo JJ, Li X, Patel NC. Utilization, price, and spending trends for antidepressants in the US Medicaid Program. Research In Social & Administrative Pharmacy 2008; 4(3):244-257.

15. Marques-Vidal P, Montaye M, Ruidavets JB, Amouyel P, Ferrieres J. Evolution and cost trends of antihypertensive and hypolipidaemic drug treatment in France. Cardiovascular Drugs & Therapy 2003; 17(2):175-189.

16. DiMasi JA, Hansen RW, Grabowski HG. The price of innovation: new estimates of drug development costs. Journal of Health Economics 2003; 22:151-185.

17. DiMasi JA, Feldman L, Secker A, Wilson A. Trends in risks associated with new drug development: success rates for new investigational drugs. Clinical Pharmacology & Therapeutics 2010; 87:272-277.

18. Gold ER, Kaplan W, Orbinski J, Harland-Logan S, N-Marandi S. Are patents impeding medical care and innovation. PLoS Medicine 2009; 7(1):e1000208.

19. Gregson N, Sparrowhawk K, Mauskopf J, Paul J. Pricing medicines: theory and practice, challenges and opportunities. Nature Reviews Drug Discovery 2005; 4(2):121-130.

20. Kola I. The state of innovation in drug development. Clinical Pharmacology & Therapeutics 2008; 83:227-230.

For peer review only

For peer review only - http://bmjopen.bmj.com/site/about/guidelines.xhtml

21. Owens J. 2006 drug approvals: finding the niche. Nature Reviews Drug Discovery 2007; 6:99-101.

22. Aitken, M, Kleinrock M. Global medicines use in 2020: outlook and implications. Parsippany, New Jersey, USA: IMS Institute for Healthcare Informatics; November 2015.

23. World Health Organization (WHO). International statistical classification of diseases and related health problems - 10th revision (ICD-10), Fifth edition, 2016. http://apps.who.int/classifications/icd10/browse/2016/en Accessed 08 July 2018.

24. European Medicine Agency (EMA). http://www.ema.europa.eu/ema/ accessed 20 July 2016.

25. Medicines & Healthcare products Regulatory Agency. https://www.gov.uk/government/organisations/medicines-and-healthcare-products-regulatory-agency Accessed 23 June 2016.

26. British National Formula (BNF). BNF Editions 57-65 (March 1981- September 2015). British Medical Association; Royal Pharmaceutical Society of Great Britain. http://www.bnf.org accessed 19 May 2018.

27. Datapharm. http://www.datapharm.org.uk/ Accessed 19July 2016.

28. Adis. http://www.springer.com/gp/adis Accessed 18 July 2016.

29. UK PharmaScan. www.ukpharmascan.org.uk accessed 22 May 2016.

30. National Institute for Health and Care Excellence (NICE) Guide to the process of technology appraisal. Published 2 September 2014. http://www.nice.org.uk/article/pmg19/resources/non-guidance-guide-to-the-processes-of-technology-appraisal-pdf accessed 11 May 2017.

31. HM Treasury. GDP deflators at market prices, and money GDP. London, UK, HM Treasury. https://www.gov.uk/government/collections/gdp-deflators-at-market-prices-and-money-gdp Accessed 11 May 2017.

For peer review only

32. Lu ZJ, Comanor WS. Strategic pricing of new pharmaceuticals. Review of Economics and Statistics, 1998; 80(1):108-118.

33. Mazzucato M. High cost of new drugs: Why government must negotiate a better deal for publicly funded research. BMJ 2016; 354:i4136.

34. The Office for Health Economics (OHE). https://www.ohe.org/news/ohe-study-pharmaceutical-rd-costs-released Accessed 27 March 2017.

35. Howard DH, Bach PB, Berndt ER, Conti RM. Pricing in the market for anticancer drugs. Journal of Economic Perspectives 2015; 29(1):139-162.

36. Hartmann M. Impact assessment of the European Clinical Trials Directive: a longitudinal, prospective, observational study analyzing patterns and trends in clinical drug trial applications submitted since 2001 to regulatory agencies in six EU countries. Trials 2012; 13:53.

37. Ward DJ, Hammond E, Linden-Phillips L, et al. Trends in clinical development timeframes for antiviral drugs launched in the UK, 1981–2014: a retrospective observational study. BMJ Open 2015; 5:e009333.

38. Munos B. Lessons from 60 years of pharmaceutical innovation. Nature Reviews Drug Discovery 2009; 8:959-68.

39. Scannell JW, Blanckley A, Boldon H, et al. Diagnosing the decline in pharmaceutical R&D efficiency. Nature Reviews Drug Discovery 2012; 11:191–200.

40. Hilner BE, Smith TJ. Efficacy does not necessarily translate to cost effectiveness: a case study in the challenges associated with 21st-century cancer drug prices. J Clin Oncol 2009; 27:2111-3.

41. Aronson JK, Ferner RE, Hughes DA. Defining rewardable innovation in drug therapy. Nature Reviews Drug Discovery 2012; 11:253–4.

For peer review only

42. Ward DJ, Slade A, Genus T, et al. How innovative are new drugs launched in the UK? A retrospective study of new drugs listed in the British National Formulary (BNF) 2001–2012. BMJ Open 2014; 4:e006235.

For peer review only

For peer review only - http://bmjopen.bmj.com/site/about/guidelines.xhtml

Figure 1. Inflation-adjusted 28-day launch price of newly licensed drugs and new licensed indications for existing marketed drugs for one of five selected conditions, 1981 to 2015 (inclusive).

Figure 2. Relative (percent, %) change in inflation-adjusted 28-day launch price of follow-on drugs compared to first of kind drugs by number of years since launch of first of kind.

For peer review only

For peer review only - http://bmjopen.bmj.com/site/about/guidelines.xhtml

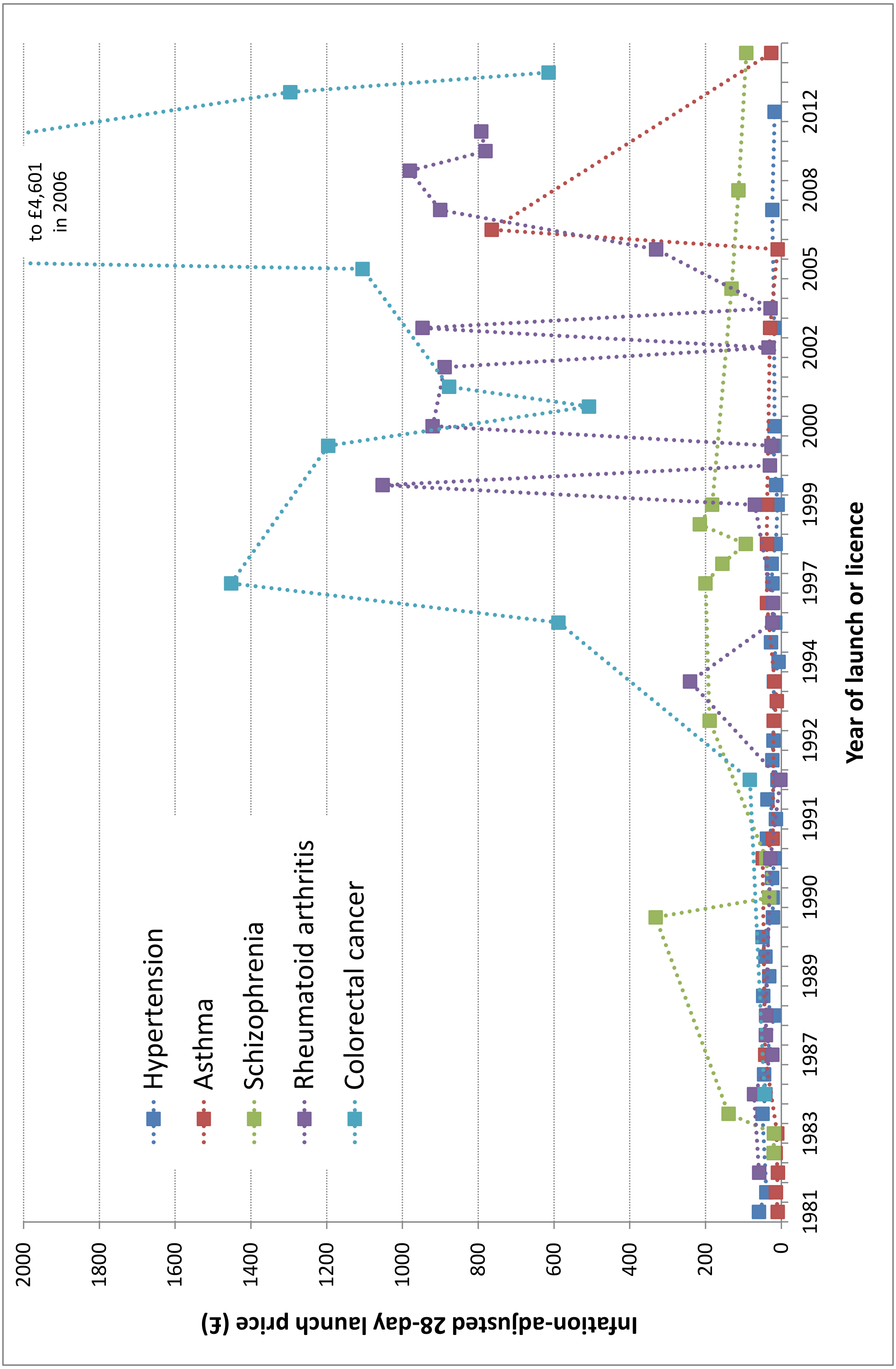
Hypertension
Asthma
Schizophrenia
Rheumatoid arthritis
Colorectal cancer
to £4,601 in 2006
Inflation-adjusted 28-day launch price (£)
2000
1800
1600
1400
1200
1000
800
600
400
200
0
Year of launch or licence
1981
1983
1987
1989
1990
1991
1992
1994
1997
1999
2000
2002
2005
2008
2012

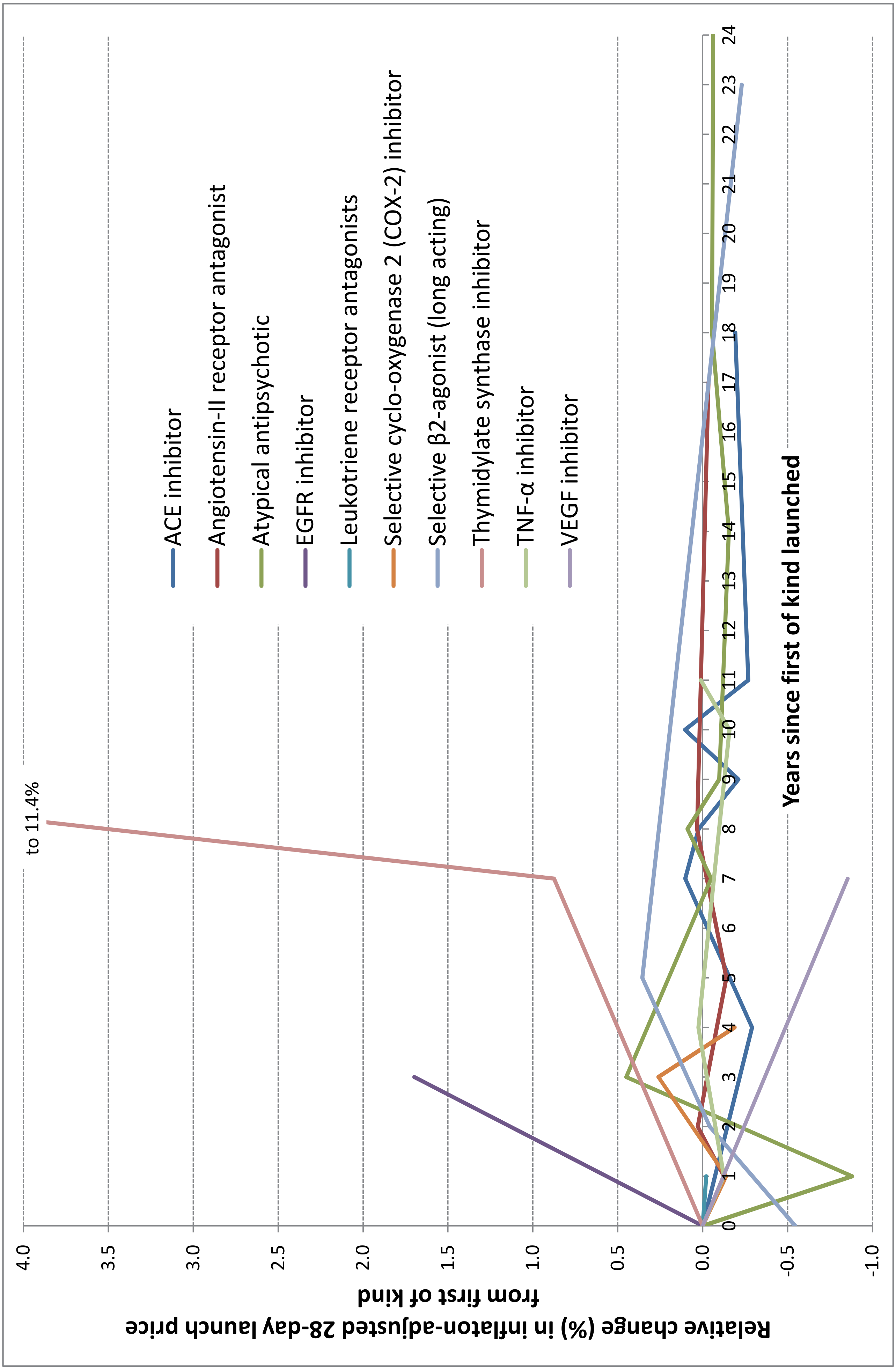
ACE inhibitor
Angiotensin-II receptor antagonist
Atypical antipsychotic
EGFR inhibitor
Leukotriene receptor antagonists
Selective cyclo-oxygenase 2 (COX-2) inhibitor
Selective β2-agonist (long acting)
Thymidylate synthase inhibitor
TNF-α inhibitor
VEGF inhibitor
to 11.4%
Years since first of kind launched
Relative change (%) in infiaton-adjusted 28-day launch price from first of kind
4.0
3.5
3.0
2.5
2.0
1.5
1.0
0.5
0.0
-0.5
-1.0
0
1
2
3
4
5
6
7
8
9
10
11
12
13
14
15
16
17
18
19
20
21
22
23
24

Disease area	Drug	Class/Mechanism	Year of launch	Adjusted 28-day launch price (£)	First of kind	Biologic drug	New drug or new indication	Notes
Hypertension	Captopril	ACE inhibitor	1981	59.06	Yes	No	New drug	
Hypertension	Indoramin	α1-adrenergic receptor antagonist	1981	39.37	No	No	New drug	
Hypertension	Dilitiazem	Calcium channel blocker	1984	49.82	No	No	New drug	
Hypertension	Enalapril	ACE inhibitor	1985	41.85	No	No	New drug	
Hypertension	Nicardipine	Calcium channel blocker	1986	45.52	No	No	New drug	
Hypertension	Terazosin	α1-adrenergic receptor antagonist	1987	38.01	No	No	New drug	
Hypertension	Bisoprolol fumarate	β-adrenergic receptor antagonist	1988	19.29	No	No	New drug	
Hypertension	Lisinopril	ACE inhibitor	1988	47.91	No	No	New drug	
Hypertension	Doxazosin	α1-adrenergic receptor antagonist	1989	32.02	No	No	New drug	
Hypertension	Isradipine	Calcium channel blocker	1989	41.86	No	No	New drug	
Hypertension	Quinapril	ACE inhibitor	1989	49.27	No	No	New drug	
Hypertension	Amlodipine	Calcium channel blocker	1990	22.05	No	No	New drug	
Hypertension	Perindopril	ACE inhibitor	1990	22.57	No	No	New drug	
Hypertension	Ramipril	ACE inhibitor	1990	24.45	No	No	New drug	
Hypertension	Celiprolol	β-adrenergic receptor antagonist	1991	17.13	No	No	New drug	
Hypertension	Cilazapril	ACE inhibitor	1991	37.74	No	No	New drug	
Hypertension	Felodipine	Calcium channel blocker	1991	14.20	No	No	New drug	
Hypertension	Fosinopril	ACE inhibitor	1991	36.70	No	No	New drug	
Hypertension	Esmolol	β-adrenergic receptor antagonist	1992	9.93	No	No	New drug	
Hypertension	Lacidipine	Calcium channel blocker	1992	23.73	No	No	New drug	
Hypertension	Trandolapril	ACE inhibitor	1992	20.86	No	No	New drug	
Hypertension	Carvedilol	β-adrenergic receptor antagonist	1994	19.28	No	No	New drug	
Hypertension	Torasemide	Loop diuretic	1994	7.41	No	No	New drug	
Hypertension	Losartan	Angiotensin-II receptor antagonist	1995	27.26	Yes	No	New drug	
Hypertension	Moxonidine	Imidazoline I_1 receptor agonist	1996	15.82	Yes	No	New drug	
Hypertension	Valsartan	Angiotensin-II receptor antagonist	1996	23.85	No	No	New drug	
Hypertension	Candesartan	Angiotensin-II receptor antagonist	1997	23.24	No	No	New drug	
Hypertension	Irbesartan	Angiotensin-II receptor antagonist	1997	25.41	No	No	New drug	
Hypertension	Lercanidipine	Calcium channel blocker	1998	14.77	No	No	New drug	
Hypertension	Imidapril	ACE inhibitor	1999	9.51	No	No	New drug	
Hypertension	Nebivolol	β-adrenergic receptor antagonist	1999	13.91	No	No	New drug	
Hypertension	Eprosartan	Angiotensin-II receptor antagonist	2000	20.82	No	No	New drug	
Hypertension	Telmisartan	Angiotensin-II receptor antagonist	2000	17.78	No	No	New drug	
Hypertension	Olmesartan	Angiotensin-II receptor antagonist	2003	18.68	No	No	New drug	
Hypertension	Aliskiren	Renin inhibitor	2007	23.71	Yes	No	New drug	
Hypertension	Azilsartan medoxomil	Angiotensin-II receptor antagonist	2012	17.73	No	No	New drug	
Asthma	Reproterol hydrochloride aerosol inhalation	Selective β2-agonist (short acting)	1981	9.22	No	No	New drug	
Asthma	Reproterol hydrochloride tablets	Selective β2-agonist (short acting)	1981	14.20	No	No	New drug	
Asthma	Hydrocortisone acetate	Corticosteroid	1982	8.64	No	No	New indication	
Asthma	Budesonide	Corticosteroid	1983	14.59	No	No	New drug	
Asthma	Pirbuterol aerosol inhalation	Selective β2-agonist (short acting)	1983	10.93	No	No	New drug	
Asthma	Nedocromil sodium	Cromoglicate	1987	42.74	No	No	New drug	
Asthma	Salmeterol	Selective β2-agonist (long acting)	1991	49.22	Yes	No	New drug	
Asthma	Tulobuterol hydrochloride	Selective β2-agonist (long acting)	1991	22.37	No	No	New drug	
Asthma	Bambuterol hydrochloride	Selective β2-agonist (long acting)	1993	20.27	No	No	New drug	
Asthma	Pirbuterol tablets	Selective β2-agonist (short acting)	1993	12.13	No	No	New drug	
Asthma	Fluticasone propionate	Corticosteroid	1994	17.53	No	No	New drug	
Asthma	Formoterol fumarate	Selective β2-agonist (long acting)	1996	37.77	No	No	New drug	
Asthma	Montelukast	Leukotriene receptor antagonists	1998	37.97	Yes	No	New drug	
Asthma	Zafirlukast	Leukotriene receptor antagonists	1999	37.19	No	No	New drug	
Asthma	Mometasone furoate	Corticosteroid	2003	29.74	No	No	New indication	
Asthma	Ciclesonide	Corticosteroid	2006	9.67	No	No	New drug	
Asthma	Omalizumab	Anti IgE humanised monoclonal antibody	2006	764.45	Yes	Yes	New drug	
Asthma	Fluticasone furoate and vilanterol	Selective β2-agonist (long acting)	2014	26.43	No	No	New drug	
Schizophrenia	Levomepromazine maleate	First generation antipsychotic	1983	20.22	No	No	New drug	
Schizophrenia	Zuclopenthixol dihydrochloride	First generation antipsychotic	1983	19.41	No	No	New drug	
Schizophrenia	Sulpiride	Atypical antipsychotic	1984	139.22	No	No	New drug	Clozapine is usually considered the first atypical antipsychotic to be launched, sulpiride was reclassified later.
Schizophrenia	Clozapine	Atypical antipsychotic	1990	331.80	Yes	No	New drug	
Schizophrenia	Loxapine	First generation antipsychotic	1990	31.75	No	No	New drug	
Schizophrenia	Remoxipride	Atypical antipsychotic	1991	39.75	No	No	New drug	
Schizophrenia	Risperidone	Atypical antipsychotic	1993	189.15	No	No	New drug	
Schizophrenia	Olanzapine	Atypical antipsychotic	1997	200.04	No	No	New drug	
Schizophrenia	Sertindole	Atypical antipsychotic	1997	155.55	No	No	New drug	
Schizophrenia	Amisulpride	Atypical antipsychotic	1998	93.95	No	No	New drug	
Schizophrenia	Quetiapine	Atypical antipsychotic	1998	214.80	No	No	New drug	
Schizophrenia	Zotepine	Atypical antipsychotic	1999	182.40	No	No	New drug	
Schizophrenia	Aripiprazole	Atypical antipsychotic	2004	131.25	No	No	New drug	
Schizophrenia	Paliperidone	Atypical antipsychotic	2008	112.92	No	No	New drug	
Schizophrenia	Lurasidone	Atypical antipsychotic	2014	92.39	No	No	New drug	
Rheumatoid arthritis	Tiaprofenic acid	Non-steroidal anti-inflammatory drug (NSAID)	1982	58.61	No	No	New drug	
Rheumatoid arthritis	Etodolac	Non-steroidal anti-inflammatory drug (NSAID)	1985	71.92	No	No	New drug	

For peer review only

Rheumatoid arthritis	Sulfasalazine	Aminosalicylate	1987	23.59	Yes	No	New indication
Rheumatoid arthritis	Nabumetone	Non-steroidal anti-inflammatory drug (NSAID)	1987	40.52	No	No	New drug
Rheumatoid arthritis	Tenoxicam	Non-steroidal anti-inflammatory drug (NSAID)	1988	40.38	No	No	New drug
Rheumatoid arthritis	Acemetacin	Non-steroidal anti-inflammatory drug (NSAID)	1991	28.64	No	No	New drug
Rheumatoid arthritis	Methotrexate	Dihydrofolate reductase inhibitor	1992	2.22	Yes	No	New indication
Rheumatoid arthritis	Ciclosporin	Calcineurin inhibitor	1994	240.63	Yes	No	New indication
Rheumatoid arthritis	Aceclofenac	Non-steroidal anti-inflammatory drug (NSAID)	1996	23.45	No	No	New drug
Rheumatoid arthritis	Meloxicam	Non-steroidal anti-inflammatory drug (NSAID)	1996	21.81	No	No	New drug
Rheumatoid arthritis	Leflunomide	Pyrimidine synthesis inhibitor	1999	69.71	Yes	No	New drug
Rheumatoid arthritis	Infliximab	TNF-α inhibitor	1999	1052.22	Yes	Yes	New indication
Rheumatoid arthritis	Rofecoxib	Selective cyclo-oxygenase 2 (COX-2) inhibitor	1999	30.12	Yes	No	New drug
Rheumatoid arthritis	Celecoxib	Selective cyclo-oxygenase 2 (COX-2) inhibitor	2000	25.97	No	No	New drug
Rheumatoid arthritis	Etanercept	TNF-α inhibitor	2000	920.27	No	Yes	New drug
Rheumatoid arthritis	Anakinra	IL-1 inhibitor	2002	888.47	Yes	Yes	New drug
Rheumatoid arthritis	Etoricoxib	Selective cyclo-oxygenase 2 (COX-2) inhibitor	2002	33.80	No	No	New drug
Rheumatoid arthritis	Adalimumab	TNF-α inhibitor	2003	946.83	No	Yes	New drug
Rheumatoid arthritis	Valdecoxib	Selective cyclo-oxygenase 2 (COX-2) inhibitor	2003	28.12	No	No	New drug
Rheumatoid arthritis	Rituximab	CD20 inhibitor	2006	330.65	Yes	Yes	New indication
Rheumatoid arthritis	Abatacept	Inhibitor of T-cell co-stimulation	2007	900.14	Yes	Yes	New drug
Rheumatoid arthritis	Tocilizumab	IL-6 inhibitor	2009	980.44	Yes	Yes	New drug
Rheumatoid arthritis	Certolizumab pegol	TNF-α inhibitor	2009	781.10	No	Yes	New drug
Rheumatoid arthritis	Golimumab	TNF-α inhibitor	2010	792.23	No	Yes	New drug
Colorectal cancer	Fluorouracil (5FU)	Thymidylate synthase inhibitor	1985	44.26	Yes	No	New indication
Colorectal cancer	Folinic acid + 5FU	Thymidylate synthase inhibitor	1992	83.04	No	No	New indication
Colorectal cancer	Raltitrexed	Thymidylate synthase inhibitor	1996	588.05	No	No	New drug
Colorectal cancer	Irinotecan	DNA topoisomerase I inhibitor	1997	1452.13	Yes	No	New drug
Colorectal cancer	Oxaliplatin	Platinum-based chemotherapeutic drug	2000	1195.80	Yes	No	New drug
Colorectal cancer	Capecitabine	Thymidylate synthase inhibitor	2001	507.49	No	No	New drug
Colorectal cancer	Tegafur (with uracil)	Thymidylate synthase inhibitor	2001	876.52	No	No	New drug
Colorectal cancer	Cetuximab	EGFR inhibitor	2005	1105.20	Yes	Yes	New drug
Colorectal cancer	Bevacizumab	VEGF inhibitor	2006	4200.67	Yes	Yes	New drug
Colorectal cancer	Panitumumab	EGFR inhibitor	2008	2981.95	No	Yes	New drug
Colorectal cancer	Regorafenib	Multi-tyrosine kinase inhibitor	2013	1296.13	Yes	No	New drug
Colorectal cancer	Aflibercept	VEGF inhibitor	2013	614.11	No	Yes	New drug

For peer review only

BMJ Open

Trends in the costs of drugs launched in the United Kingdom between 1981 and 2015: an analysis of the launch price of drugs in five disease areas

Journal:	*BMJ Open*
Manuscript ID	bmjopen-2018-027625.R1
Article Type:	Research
Date Submitted by the Author:	06-Feb-2019
Complete List of Authors:	Ward, Derek; University of Birmingham, Institute of Applied Health Reseach<br>Doos, Lucy; University of Birmingham, College of Medical and Dental Sciences<br>Stevens, Andrew; University of Birmingham, College of Medical and Dental Sciences
**Primary Subject Heading**:	Health policy
Secondary Subject Heading:	Pharmacology and therapeutics
Keywords:	Drug pricing, Trends, pharmaceutical innovation, United KIngdom

SCHOLARONE™
Manuscripts

Trends in the costs of drugs launched in the United Kingdom between 1981 and 2015: an analysis of the launch price of drugs in five disease areas

Derek J Ward[1], Lucy Doos[2], Andrew Stevens[1].

1. Institute of Applied Health Research
   University of Birmingham, Birmingham, United Kingdom
2. College of Medical and Dental Sciences
   University of Birmingham, Birmingham, United Kingdom

Corresponding author:

Dr Derek Ward d.j.ward@bham.ac.uk Tel. 0121 414 7660

Murray Learning Centre, Institute of Applied Health Research, University of Birmingham, Edgbaston, Birmingham, B15 2TT, United Kingdom

Word count (excluding title page, abstract, references, figures and tables): 3,390

Keywords: drug pricing, trends, pharmaceutical innovation, United Kingdom

For peer review only

ABSTRACT

Objectives: To investigate the trend in launch price of new drugs for five common health conditions.

Design: Cross-sectional study using data on new drugs launched in the UK between 1981 and 2015 for hypertension, asthma, rheumatoid arthritis, schizophrenia and colorectal cancer.

Data and sources: All drugs marketed in the UK between 1981 and 2015 (inclusive), and licensed specifically for the treatment of one of the five chosen conditions were included in the study. Newly launched medicines and their launch prices were identified by hand-searching all editions of the British National Formulary (BNF) in addition to searching the websites of relevant regulatory agencies (European Medicines Agency and Medicines and Healthcare products Regulatory Agency). The launch price in UK pounds for a 28-day supply of each medicine at a typical or usual maintenance dose was adjusted for the effects of general inflation using the Gross Domestic Product (GDP) deflator series.

Results: 104 drugs were included in our study with a mean inflation adjusted 28-day launch price of £288 (SD £678). The launch price of new drugs varied significantly across the five conditions, with drugs for hypertension having the lowest mean price (£27) and drugs for colorectal cancer having the highest mean price (£1,590) (p<0.001). There were large increases in launch prices across the study period, but the magnitude and pattern was markedly different between therapeutic areas. Biologic drugs represented 13.5% of all included drugs and had a significantly higher launch price than non- biologic drugs (£1,233 vs £141, p<0.001). 22.1% of included drugs were first of kind and had a significantly higher launch price than follow-on drugs (£768 vs £151) (p<0.0001).

For peer review only

Conclusion: Drugs prices continue to increase across different therapeutic areas. This has some association with novelty, but, it is not clear if this increase in price is associated with medical benefits.

For peer review only

For peer review only - http://bmjopen.bmj.com/site/about/guidelines.xhtml

ARTICLE SUMMARY

Strengths and limitations of this study

- The timeline of this study enables a very long-term view of drug pricing that goes beyond previous published work.
- This study used the British National Formulary to identify new drugs and new licensed indications for existing marketed drugs and is therefore likely to represent a comprehensive view of drug pricing in the UK.
- This study is restricted to publicly available pricing data in the UK; the actual price paid by healthcare providers for drugs may vary from this and the results may not be applicable to other settings.
- This study chose to focus on five health conditions in a pragmatic way, and the results may not be generalisable to drugs licensed for use in other health conditions.

For peer review only

For peer review only - http://bmjopen.bmj.com/site/about/guidelines.xhtml

BACKGROUND

Over last few decades, spending on healthcare has risen faster than economic growth in many developed countries.[1;2] Internationally, spending on pharmaceuticals represents a significant proportion of the total healthcare budget.[3] For example, high-income countries within the Organisation for Economic Co-operation and Development (OECD) spend on average 18% of their total healthcare expenditure on medicines and this figure can reach up to 80% in some low- and middle-income countries.[3] In the UK, the expenditure on medicines represented 11.6% of total healthcare expenditure in 2008.[1]

Worldwide, affordability is a major component of ensuring access to essential medicines for many conditions.[4,5] Affordability reflects both price and volume, and many publicly funded healthcare providers, including the UK National Health Service (NHS), aim to provide effective treatment at a price that represents value for money.[6] Healthcare systems in many countries use a variety of cost-saving and cost-containing measures in order to counter financial challenges, including, in the UK, a Government wide agreement with industry to cap increases in overall expenditure on branded medicines (the Pharmaceutical Price Regulation Scheme) and ensuring approval and reimbursement of drugs is dependent on an assessment of clinical and cost effectiveness using Health Technology Assessment (HTA), which may include restrictions on patient eligibility.[6] An understanding of the drivers of medicine prices is therefore important particularly where countries and policy makers are seeking to develop pricing policies that improve both the availability and the affordability of such medicines.[3]

The increasing cost of pharmaceuticals used to manage a number of common conditions has received increasing attention in recent years.[4,7] In the USA, retail prescription drug spending accelerated in 2014, growing 13.1% in one year, representing the largest annual increase since

For peer review only

2003.[8] According to a recent report that considered the impact of changes in the pharmaceutical industry and its impact on healthcare payers in the USA[8], this increase was the result of increasing demand and changes in patient behaviour, which both had a significant impact on drug expenditures.

However, innovation or novelty is also a factor, as pricing of first of kind drugs was noted to be one of the most important factors driving this trend.[8] Studies on rising overall pharmaceutical expenditure seen during the 1990s to mid-2000s in North America and Europe have highlighted the role played by both increased utilisation and the adoption of newer, more expensive medicines.[9,10] The high cost of newer agents has been identified by some commentators as the key component of rising per-patient pharmaceutical costs for cancer chemotherapy and the treatment of diabetes mellitus, glaucoma, psychosis, multiple sclerosis and haemophilia.[11-12] However, other studies suggest increasing utilisation as the major driver of rising costs in the treatment of hypertension, hyperlipidaemia, depression, and rheumatoid arthritis, where increasing overall pharmaceutical spending has not necessarily been accompanied by increased per-patient spending.[13-15]

Increases in the cost of newer medicines has been variously explained as due to increasing regulatory and technology assessment requirements, research and development costs, attrition rates, production costs, the patent system, and marketing practice.[16-22] To further investigate trends in the price of pharmaceuticals, we sought to describe long-term, 35-year trends in the launch price for all new medicines marketed in the UK to treat one of five common conditions.

For peer review only

METHODS

Selection of disease areas and identification of new drugs

All medicines marketed in the UK for the first time between 1981 and 2015 (inclusive) for the treatment of one the following five conditions were included in the study using International Statistical Classification of Diseases version 10 (ICD-10)[23] : 1) Asthma (ICD-10 code J45), 2) Colorectal cancer (ICD-10 code C18), 3) Essential hypertension (ICD-10 code I10), 4) Rheumatoid arthritis (seropositive rheumatoid arthritis [ICD-10 codes M05 and M06]), and 5) Schizophrenia (ICD-10 code F20). These diseases were selected by consensus of the six members of the NIHR Horizon Scanning Research and Intelligence Centre research group to represent a range of physical and mental health disorders for which significant pharmaceutical innovation over the period of interest was known to have occurred.

Our study included medicines specifically licensed for the treatment of one of these conditions at the time of first UK launch (as described in the Summary of Product Characteristics [SmPC] published by the European Medicines Agency [EMA][24] and Medicines and Healthcare products Regulatory Agency [MHRA][25], as appropriate) as well as existing licensed drugs that subsequently received Marketing Authorisation for one of the five study conditions, including those that have subsequently been withdrawn.

Medicines were identified by hand-searching all editions of the British National Formulary (BNF)[26] published between 1981 and 2015. The BNF lists all medicines available for prescription and dispensing within the UK irrespective of whether they are available on the NHS; the date of first appearance in the BNF (for new medicines) or the date where the indication was amended in the BNF (for existing licensed medicines subsequently receiving Marketing Authorisation for one of the specified conditions) was taken to be the year of launch in the UK. Medicines listed in BNF edition 1

For peer review only - http://bmjopen.bmj.com/site/about/guidelines.xhtml

were considered to already be available for use in 1981, and therefore excluded from the study. This approach to identifying all relevant new medicines was supplemented by searches of the electronic Medicines Compendium (eMC [Datapharm Communications Ltd.])[27], commercial pharmaceutical databases (Adis Insight [Springer][28] and Pharmaprojects [Informa Healthcare])[29], the websites of relevant regulatory agencies (EMA and MHRA)[24,25], and searches for clinical practice guidelines issued by the National Institute for Health and Clinical Excellence (NICE)[30] and relevant specialist clinical societies and associations.

We had no patient or public involvement as part of this study.

Price calculation

To allow price comparisons within each disease area, the launch price in UK pounds for a 28-day supply of each medicine at a typical or usual maintenance dose (including cycles or courses where relevant) was calculated from the unit price provided in the edition of the BNF in which the medicine first appeared. Initial loading or lower introductory doses were ignored. The published SmPC and/or relevant clinical guidelines were consulted where no usual dose was listed in the BNF. The dose range mid-point was used where no usual dose could be established (adjusted to be achievable using available dose formulations). In all cases, the price was calculated using the least expensive combination of available dose formulations (where relevant, this assumed wastage, a body weight of 76.9kg, and body surface area of 1.7m^2).

The 28-day launch prices for all medicines were adjusted for the effects of general inflation using the Gross Domestic Product (GDP) deflator series.[31] GDP deflators serve as a measure of inflation within the economy as a whole for a given time period and were used to adjust prices to 2015 values, thereby allowing direct comparisons between prices at different time points.

For peer review only - http://bmjopen.bmj.com/site/about/guidelines.xhtml

We excluded new combinations of existing licensed medicines and those not found in the BNF.

Data handling and analysis

Data on drug name, whether it was newly launched or an existing licensed and available product, the mechanism of action or drug class, whether the drug was a biological agent, launch year, launch price for a typical 28-day supply and 2015 adjusted 28-day launch price were extracted and entered onto an Excel spreadsheet. Where the mechanism of action or drug class was not stated in the BNF, it was obtained from the commercial Pharmaprojects database. Statistical analysis was carried out using IBM SPSS (version 21) for Windows. Trends in adjusted 28-day launch prices were initially explored using simple descriptive statistics and scatter plots. Descriptive analyses were presented as means and standard deviations (SD) for normally distributed continuous variables. Significant differences were determined using ANOVA for continuous normally distributed data and X^2 for dichotomous variables.

For peer review only

RESULTS

104 drugs met our inclusion criteria, representing approximately 10% of all new drugs launched for all conditions over the period of the study.[32] They were launched with an inflation-adjusted 28-day price that ranged from £2.20 (methotrexate for rheumatoid arthritis, 1992) to £4,200.70 (bevacizumab for colorectal cancer, 2006) (Table 1). Nearly one third of the drugs were launched for hypertension (34.6%), while just 10.6% were launched for colorectal cancer. There were statistically significant differences in the mean inflation-adjusted 28-day launch price by condition, with drugs for hypertension having the lowest mean price (£27) and drugs for colorectal cancer having the highest mean price (£1,590) (p<0.0001).

Drug characteristics

The majority of the drugs included in the study were newly launched (92.3%) as opposed to new licensed indications for existing marketed drugs (9.6%). The mean inflation-adjusted 28-day price for newly launched drugs was higher than that for new licensed indications for existing marketed drugs. This pattern was observed across all three relevant conditions (asthma, rheumatoid arthritis and colorectal cancer), but the differences were not statistically significant (Table 2).

Fourteen (13.5%) of the included drugs were biologic agents, almost two thirds of which were for rheumatoid arthritis, and none of which were for hypertension or schizophrenia. Over the three relevant conditions, biologic drugs had a significantly higher mean inflation-adjusted 28-day launch price than non-biologic drugs (£1,233 vs £141, p<0.001).

Newly launched biologics had nearly twice the mean inflation-adjusted 28-day launch price of the two newly licensed indications for existing marketed biologic drugs (£1322 vs £691), however this difference was not statistically significant.

For peer review only

Table 1: Number of drugs and their 28-day inflation-adjusted launch price by selected condition launched in the period 1981 to 2015 (inclusive)

Includes newly licensed drugs as well as new licensed indications for existing marketed drugs. Prices adjusted to 2015 values using the Gross Domestic Product (GDP) deflator series.

Selected condition	Number (%)	Mean price (SD)	Median price (range)
Hypertension	36 (34.6%)	£27.01 (13.09)	£23.48 (7.40-59.1)
Asthma	18 (17.3%)	£64.73 (175.10)	£21.32 (8.60-764.50)
Schizophrenia	15 (14.4%)	£130.31 (86.56)	£131.25 (19.40-331.80)
Rheumatoid arthritis	24 (23.1%)	£347.16 (414.24)	£69.71 (2.20-1052.20)
Colorectal cancer	11 (10.6%)	£1,590.30 (1,426.65)	£1,105.20 (83.04-4200.70)
Total	104	£287.67 (678.34)	£37.76 (2.20-4200.70)

For peer review only

Table 2: Mean 28-day inflation-adjusted launch price by selected condition according to biologic vs non-biologic drug type, first of kind vs follow on drug, and new drug vs new indication for existed marketed drug, 1981-2015.

Selected condition	Biologic drug	Non-biologic drug	First of kind	Follow on	New drug	New licensed indication
Hypertension			£31.50	£26.50		
			P=0.47			
Asthma	£764.50	£23.60	£283.90	£20.90	£70.40	£19.20
	p<0.001		p=0.01		p=0.71	
Schizophrenia			£331.80	£115.90		
			p=0.01			
Rheumatoid arthritis	£813.90	£211.90	£451.80	£227.40	£389.70	£329.90
	p=0.001		p=0.31		p=0.79	
Colorectal cancer	£2,225.48	£1,227.35	£2,368.44	£941.86	£1,741.03	£83.04
	p=0.28		p=0.10		p=0.29	
All conditions	£1,232.76	£140.65	£768.25	£151.21	£293.19	£221.34
	p<0.001		p<0.001		p=0.78	

For peer review only

Pattern of change in pricing

A large increase in inflation-adjusted 28-day launch prices was observed when all drugs included in this study were considered together. However, the magnitude and pattern of change varied considerably between the five therapeutic areas (Figure 1).

For hypertension, following the introduction of captopril in 1981, the typical price of newly launched drugs generally fell (with some variation) until the early 1990s, after which prices appeared to stabilise. For asthma, there was a single high-cost outlier (omalizumab, launched in 2006), but there was also a sharp increase in launch price in 1987 with the launch of nedocromil sodium (a cromoglicate), which was nearly three times the inflation-adjusted price of the previously launched drug. The overall trend in the price of newly launched drugs for schizophrenia was broadly flat. However, there were some significant variations across the period of interest. In particular, two drugs launched in 1990 showed a great discrepancy in inflation-adjusted 28-day launch price, with the price of clozapine (£332.80, the first atypical antipsychotic launched) being nearly ten-fold higher than loxapine £31.75), which was launched the same year. For rheumatoid arthritis launch prices experienced a marked upward step in 1999 with the arrival of infliximab (TNF-α inhibitor), the first biologic agent licensed for this condition. This was followed by a slight decrease in the inflation-adjusted price the following year when the next drug was launched (by 12.5%). Launch prices for drugs licensed to treat colorectal cancer rose increasingly rapidly in the two decades following the mid-1990s. In particular, the introduction of bevacizumab in 2006, the second biological drug for this condition, resulted in a nearly threefold increase in the inflation-adjusted 28-day launch price compared to drugs launched in the previous few years.

Drug class/mechanism of action

For peer review only - http://bmjopen.bmj.com/site/about/guidelines.xhtml

The 104 drugs included in our study represented 34 different drug classes or mechanisms of action, 17 of which were represented only once. There were significant variations in the inflation-adjusted 28-day launch price for drugs by drug class/mechanism of action; these differences were statistically significant across all but one of the therapeutic areas (the exception being drugs for colorectal cancer) (Appendix1).

Among the 36 drugs from nine drug classes included for hypertension, α1-adrenergic receptor antagonists had the highest mean inflation-adjusted 28-day launch price (£36.50) followed by angiotensin converting enzyme (ACE) inhibitors (£35.00). For asthma, the anti IgE humanised monoclonal antibody omalizumab had the highest inflation-adjusted launch price by far (£764.45). The next most expensive was the single cromoglicate (£42.74), followed by leukotriene receptor antagonists (£37.58) and selective β2-agonists – long acting (£31.21). The lowest mean inflation-adjusted 28-day launch price was for selective β2-agonists – short acting (£11.62). For rheumatoid arthritis, nearly one third of drugs (29.2%) were non-steroidal anti-inflammatory drugs (NSAIDs), and there was a marked downward trend in their launch prices between 1985 and 1996 (mean adjusted 28-day launch price for the whole period £40.76). TNF-α inhibitors were the second common group of drugs launched for rheumatoid arthritis, and these had a mean inflation-adjusted launch price of £898.53, though single examples of an IL-1 inhibitor, an inhibitor of T-cell co-stimulation and IL-6 inhibitors were priced even higher. Almost one third of all new drugs launched for colorectal cancer were thymidylate synthase inhibitors, which had a mean inflation-adjusted 28-day launch price £513.78. The launch price for this class of drugs showed a dramatic more than ten-fold increase between 1992 and 2001, but even so the mean launch price was still the lowest of all drug classes/mechanisms of action identified for this condition. Drugs for schizophrenia were grouped into two broad categories, recognising the difficulty of ascribing a specific class or individual mechanism of action. Atypical antipsychotics had significantly higher inflation-adjusted 28-day launch price (£156.93) than first generation antipsychotics (£23.80) ($p<0.01$).

For peer review only - http://bmjopen.bmj.com/site/about/guidelines.xhtml

First of kind and follow-on drugs

Less than a quarter of the drugs included in our study (22.1%) represented the first of a new class of drugs or a new mechanism of action (first of kind). However, these drugs had a significantly higher inflation-adjusted 28-day launch price than follow-on drugs (£768 vs £151, $p<0.0001$) (Table 2). However, within therapy areas a statistically significant difference between first of kind and follow-on drugs was only observed for asthma and schizophrenia (both $p<0.01$). First of kind drugs represented almost half of all newly launched drugs for colorectal cancer, a proportion that was greater than for any other indication.

We identified nine examples where the first of kind and at least one follow-on drug were launched in the time period of this study; two each of these drug classes/mechanisms of action were licensed for colorectal cancer, asthma, hypertension and rheumatoid arthritis, and one was licensed for schizophrenia. In general, it was apparent that inflation-adjusted launch prices generally fell over time, particularly as the time from when the first of kind was launched increased (Figure 2). The overall decrease in inflation-adjusted 28-day launch price from the first of kind to the final launch of a follow on varied between -£2.00 (selective cyclo-oxygenase 2 inhibitors for rheumatoid arthritis) to -£259.99 (TNF-α inhibitors for rheumatoid arthritis), and in relative terms varied between -6.6% (selective cyclo-oxygenase 2 inhibitors for rheumatoid arthritis) to -83.9% (ACE inhibitors for hypertension). The exception were the two EGFR inhibitors for colorectal cancer, where panitumumab was priced over 160% higher than cetuximab, the first of kind.

For peer review only - http://bmjopen.bmj.com/site/about/guidelines.xhtml

DISCUSSION

Main findings

Our findings show that while the launch prices of drugs have generally increased over the study period, after controlling for inflation there were marked differences according to therapeutic area, with some showing no real-terms increase. In addition, the launch price of first of kind drugs is consistently higher than that of other drugs, and that biological agents are considerably more expensive at launch that other drugs marketed for the same condition.

Pharmaceutical companies argue that the price of new drugs is set at a level needed to recoup the vast costs incurred in bringing that drug to market, as well as fund future research and development.[33-35] These costs have risen over time for a number of reasons, including the increasing complexity and length of clinical trial programmes to meet the needs of regulators and HTA bodies,[36-39] so that by some estimates, the rate of new drug launches per R&D spend has fallen by around a half every nine years since the 1950s.[40] However, R&D costs per drug candidate are not transparent for commercial reasons, and the oft quoted cost of bringing a new drug to market may fail to properly account for publicly funded basic and translational research.[34] In our study, the mean inflation-adjusted 28-day launch price for drugs indicated for colorectal cancer was almost 59 times greater than the price of those for hypertension. We cannot directly account for different drug development costs across different disease areas, but this finding may reflect the headroom for therapeutic gain in colorectal cancer when compared to hypertension. Our data appears to agree with the general observation made by commentators that managing cancer is expensive and the prices for drugs associated with cancer represent a great burden to healthcare systems.[36,41] Howard et al[36] reported that the average adjusted launch price of anticancer drugs increased by 10% annually between 1995 and 2013, which is considerably less than the increase in inflation-adjusted launch price of drugs for colorectal cancer we observed in the period 1985 to 2013. Launch prices spiked in 2006 with the introduction of the biological drug, bevacizumab, which might be expected

For peer review only - http://bmjopen.bmj.com/site/about/guidelines.xhtml
For peer review only

given the increased development and manufacturing costs associated with biological agents.[34] This pattern of increased drug costs associated with the introduction of the first biological drug mirrored that seen in rheumatoid arthritis, but unlike rheumatoid arthritis, commentators have argued that this pressure on prices seen in cancer is not related to the magnitude of expected health benefit.[36]

We also found that first of kind drugs were more expensive at launch and that where our data included both first of kind and follow on drugs, the price of subsequent launches was lower but still rather closely related to that of the first launch. This pattern is readily explained by the market into which new and follow on drugs are launched, with first of kind drugs competing with established drugs for the same condition or creating a new market if no equivalent treatment exists. The premium given to first of kind drugs could be justified if payers are willing to pay more to receive a substantial therapeutic advance compared to existing drug or other treatment options.[33] In our data, this may partly explain the very marked differences between areas where there is limited scope for new drugs to markedly improve care. The idea of 'rewardable innovation' suggests that health services and patients place value on improvements in a wide range of domains, including those related to new molecular structures, drug synthesis, pharmacodynamics, pharmacokinetics, drug delivery, pharmacogenetics and application in clinical practise.[42] However, many newly launched drugs do not offer any therapeutic advance for patients[41] and even many first of kind drugs do not provide any clinically significant innovation.[43]

Strengths and limitations of the study

We believe our study includes all drugs newly launched in the UK for the five indicated conditions over a 35 year period. However, we chose the five conditions on a pragmatic basis, and generalisability from our findings cannot be assumed, particularly in drugs for rare or orphan conditions, which were not part of our study. In addition, the published list price for drugs may not be actual price paid by NHS organisations, particularly for the more recently launched drugs, as this

For peer review only - http://bmjopen.bmj.com/site/about/guidelines.xhtml

fails to account for the effects of local price negotiations and the increasing use of patient access schemes. These predominate in new cancer drugs, and tend to be of an order sufficient to yield a cost-utility ratio acceptable to NICE, but their impact would tend to blunt the apparent increase in price over time for a particular condition, whereas our data still show a marked increase in the inflation-adjusted price of new drugs for colorectal cancer. Finally, we acknowledge that our decision to standardise comparisons on the basis of a 28-day supply of medicines could be criticised as failing to note that treatment for some conditions and with some medications could potentially be lifelong, and hence generate much higher total costs for payers. Indeed, some innovations in cancer could be life-extending, and so overall costs could escalate further, albeit with important health gains. However, our principal comparisons were within disease areas, and in general, this means treatments are given for a similar duration or course.

Conclusions

While prices for new drugs continue to increase overall, this masks significant disparities between therapeutic areas. It is unclear to what extent these patterns represent developers passing on real differences in development and manufacturing costs, or different pricing strategies based on an assessment of the current market and payers willingness to pay more for some conditions. Increased prices could be justified where new drugs meet an unmet clinical need [42,43] and could encourage developers to focus efforts in priority fields, where higher risk might reasonably be rewarded by higher returns. Publicly funded healthcare systems demand value for money, but existing pricing systems lack the transparency to ensure access to drugs at reasonable and justified prices.

For peer review only - http://bmjopen.bmj.com/site/about/guidelines.xhtml

CONTRIBUTION OF THE AUTHORS

DJW and AJS conceived the original study idea, and LD contributed to the development of the study design and methods. LD extracted the data, which was independently reviewed by DJW. LD and DJW conducted the analyses. All authors were involved in the interpretation of the results. LD produced the initial draft of the paper, which was then circulated repeatedly to all authors for critical revision. LD, DJW and AJS read and approved the final version. All authors had full access to all of the study data (including statistical reports and tables), and can take responsibility for the integrity of the data and the accuracy of the analysis. DJW is the guarantor.

COMPETING INTERESTS

All authors have completed the ICMJE uniform disclosure form at www.icmje.org/coi_disclosure.pdf and declare: all authors have financial support from the National Institute for Health Research (NIHR) for the submitted work; all authors have no financial relationships with any organisations that might have an interest in the submitted work in the previous three years; and all authors have no other relationships or activities that could appear to have influenced the submitted work.

FUNDING STATEMENT

The study was undertaken as part of the research programme of the NIHR Horizon Scanning Research & Intelligence Centre (NIHR HSRIC). HSRIC is funded by the National Institute for Health Research (NIHR). The NIHR had no role in the study design; in the collection, analysis and interpretation of the data; in the preparation of the manuscript; or in the decision to submit the article for publication. This article presents independent research funded by the NIHR. The views expressed are those of the authors and not necessarily those of the NHS, the NIHR or the Department of Health.

For peer review only

DATA SHARING STATEMENT

All data are included in the supplementary file.

For peer review only

For peer review only - http://bmjopen.bmj.com/site/about/guidelines.xhtml

REFERENCES

1. Organisation for Economic Co-operation and Development. OECD health data 2011. 30 June 2011. Paris, France, OECD.

2. Squires DA, The Commonwealth Fund. Explaining the high health care spending in the United States: an international comparison of supply, utilization, prices, and quality. 1595 Vol. 10. 2012. The Commonwealth Fund. Issues in International Health Policy.

3. Ud-Din Babar Z, Lessing C, Mace C, Bissell K. The Availability, pricing and affordability of three essential asthma medicines in 52 low- and middle-income countries. PharmacoEconomics 2013 31:1063–1082.

4. Creese A, Gasman N, Mariko M. The World Medicines Situation. Geneva: World Health Organization; 2004. http://apps.who.int/medicinedocs/en/d/Js6160e/ Accessed 19 July 2016.

5. Vogler S, Martikainen JE. Pharmaceutical pricing in Europe. In: Zaheer-Ud-Din Babar (ed): Pharmaceutical Prices in the 21st Century: Springer International Publishing Switzerland 2015.

6. Morrison EE, Webb DJ. UK Health Technology Assessment and value based pricing. In: Zaheer-Ud-Din Babar (ed): Pharmaceutical Prices in the 21st Century: Springer International Publishing Switzerland 2015.

7. Cohn J. The Milbank Quarterly 2016; 94(2):260-263.

8. The 2014 drug trend report. Workplace health and benefit. Medical Benefits 30 April 2015; 32 (8): 1-3.

9. Morgan S. Drug spending in Canada: recent trends and causes. Medical Care 2004; 42(7):635-642.

10. Morgan SG. Quantifying components of drug expenditure inflation: the British Columbia seniors' drug benefit plan. Health Services Research 2002; 37(5):1243-1266.

For peer review only

11. Halbert RJ, Zaher C, Wade S, Malin J, Lawless GD, Dubois RW. Outpatient cancer drug costs: changes, drivers, and the future. Cancer 2002; 94(4):1142-1150.

12. Schrag D. The price tag on progress--chemotherapy for colorectal cancer. New England Journal of Medicine 2004; 351(4):317-319.

13. Koskinen H, Martikainen JE, Maljanen T. Antipsychotics and antidepressants: an analysis of cost growth in Finland from 1999 to 2005. Clinical Therapeutics 2009; 31(pt 1):1469-77.

14. Chen Y, Kelton CM, Jing Y, Guo JJ, Li X, Patel NC. Utilization, price, and spending trends for antidepressants in the US Medicaid Program. Research In Social & Administrative Pharmacy 2008; 4(3):244-257.

15. Marques-Vidal P, Montaye M, Ruidavets JB, Amouyel P, Ferrieres J. Evolution and cost trends of antihypertensive and hypolipidaemic drug treatment in France. Cardiovascular Drugs & Therapy 2003; 17(2):175-189.

16. DiMasi JA, Hansen RW, Grabowski HG. The price of innovation: new estimates of drug development costs. Journal of Health Economics 2003; 22:151-185.

17. DiMasi JA, Feldman L, Secker A, Wilson A. Trends in risks associated with new drug development: success rates for new investigational drugs. Clinical Pharmacology & Therapeutics 2010; 87:272-277.

18. Gold ER, Kaplan W, Orbinski J, Harland-Logan S, N-Marandi S. Are patents impeding medical care and innovation. PLoS Medicine 2009; 7(1):e1000208.

19. Gregson N, Sparrowhawk K, Mauskopf J, Paul J. Pricing medicines: theory and practice, challenges and opportunities. Nature Reviews Drug Discovery 2005; 4(2):121-130.

20. Kola I. The state of innovation in drug development. Clinical Pharmacology & Therapeutics 2008; 83:227-230.

For peer review only

21. Owens J. 2006 drug approvals: finding the niche. Nature Reviews Drug Discovery 2007; 6:99-101.

22. Aitken, M, Kleinrock M. Global medicines use in 2020: outlook and implications. Parsippany, New Jersey, USA: IMS Institute for Healthcare Informatics; November 2015.

23. World Health Organization (WHO). International statistical classification of diseases and related health problems - 10th revision (ICD-10), Fifth edition, 2016. http://apps.who.int/classifications/icd10/browse/2016/en Accessed 08 July 2018.

24. European Medicine Agency (EMA). http://www.ema.europa.eu/ema/ accessed 20 July 2016.

25. Medicines & Healthcare products Regulatory Agency. https://www.gov.uk/government/organisations/medicines-and-healthcare-products-regulatory-agency Accessed 23 June 2016.

26. British National Formula (BNF). BNF Editions 57-65 (March 1981- September 2015). British Medical Association; Royal Pharmaceutical Society of Great Britain. http://www.bnf.org accessed 19 May 2018.

27. Datapharm. http://www.datapharm.org.uk/ Accessed 19July 2016.

28. Adis. http://www.springer.com/gp/adis Accessed 18 July 2016.

29. UK PharmaScan. www.ukpharmascan.org.uk accessed 22 May 2016.

30. National Institute for Health and Care Excellence (NICE) Guide to the process of technology appraisal. Published 2 September 2014. http://www.nice.org.uk/article/pmg19/resources/non-guidance-guide-to-the-processes-of-technology-appraisal-pdf accessed 11 May 2017.

31. HM Treasury. GDP deflators at market prices, and money GDP. London, UK, HM Treasury. https://www.gov.uk/government/collections/gdp-deflators-at-market-prices-and-money-gdp Accessed 11 May 2017.

For peer review only

32. Ward JD, Martino O, Simpson S, Stevens AJ. Decline in new drug launches: myth or reality? Retrospective observational study using 30 years of data from the UK. BMJ Open 2013; 3:e002088.

33. Lu ZJ, Comanor WS. Strategic pricing of new pharmaceuticals. Review of Economics and Statistics, 1998; 80(1):108-118.

34. Mazzucato M. High cost of new drugs: Why government must negotiate a better deal for publicly funded research. BMJ 2016; 354:i4136.

35. The Office for Health Economics (OHE). https://www.ohe.org/news/ohe-study-pharmaceutical-rd-costs-released Accessed 27 March 2017.

36. Howard DH, Bach PB, Berndt ER, Conti RM. Pricing in the market for anticancer drugs. Journal of Economic Perspectives 2015; 29(1):139-162.

37. Hartmann M. Impact assessment of the European Clinical Trials Directive: a longitudinal, prospective, observational study analyzing patterns and trends in clinical drug trial applications submitted since 2001 to regulatory agencies in six EU countries. Trials 2012; 13:53.

38. Ward DJ, Hammond E, Linden-Phillips L, et al. Trends in clinical development timeframes for antiviral drugs launched in the UK, 1981–2014: a retrospective observational study. BMJ Open 2015; 5:e009333.

39. Munos B. Lessons from 60 years of pharmaceutical innovation. Nature Reviews Drug Discovery 2009; 8:959-68.

40. Scannell JW, Blanckley A, Boldon H, et al. Diagnosing the decline in pharmaceutical R&D efficiency. Nature Reviews Drug Discovery 2012; 11:191–200.

41. Hilner BE, Smith TJ. Efficacy does not necessarily translate to cost effectiveness: a case study in the challenges associated with 21st-century cancer drug prices. J Clin Oncol 2009; 27:2111-3.

For peer review only

42. Aronson JK, Ferner RE, Hughes DA. Defining rewardable innovation in drug therapy. Nature Reviews Drug Discovery 2012; 11:253–4.

43. Ward DJ, Slade A, Genus T, et al. How innovative are new drugs launched in the UK? A retrospective study of new drugs listed in the British National Formulary (BNF) 2001–2012. BMJ Open 2014; 4:e006235.

For peer review only

Figure 1. Inflation-adjusted 28-day launch price of newly licensed drugs and new licensed indications for existing marketed drugs for one of five selected conditions, 1981 to 2015 (inclusive).

Figure 2. Relative (percent, %) change in inflation-adjusted 28-day launch price of follow-on drugs compared to first of kind drugs by number of years since launch of first of kind.

For peer review only

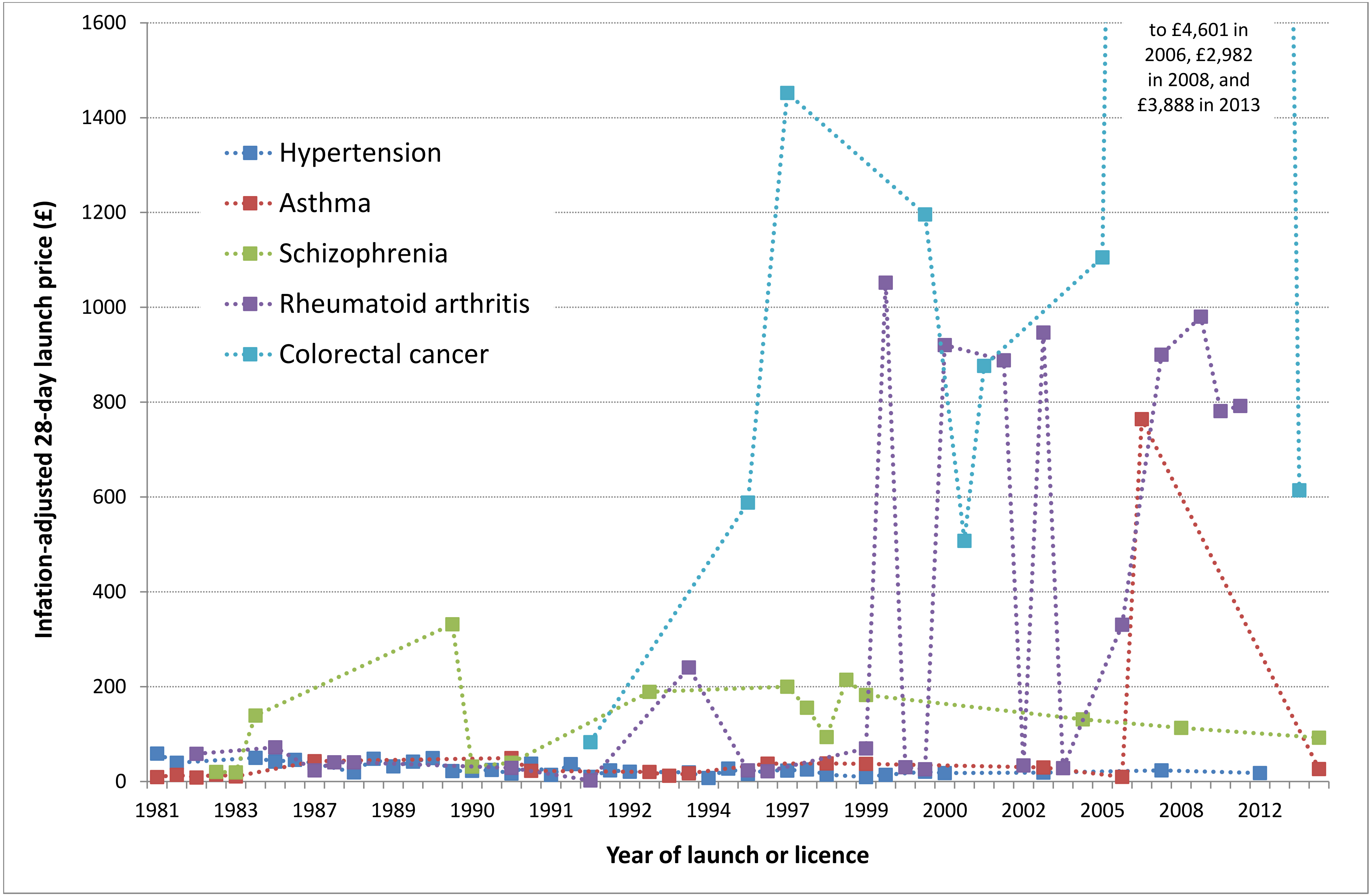
1600
1400
1200
1000
800
600
400
200
0
to £4,601 in 2006, £2,982 in 2008, and £3,888 in 2013
Hypertension
Asthma
Schizophrenia
Rheumatoid arthritis
Colorectal cancer
Inflation-adjusted 28-day launch price (£)
1981
1983
1987
1989
1990
1991
1992
1994
1997
1999
2000
2002
2005
2008
2012
Year of launch or licence

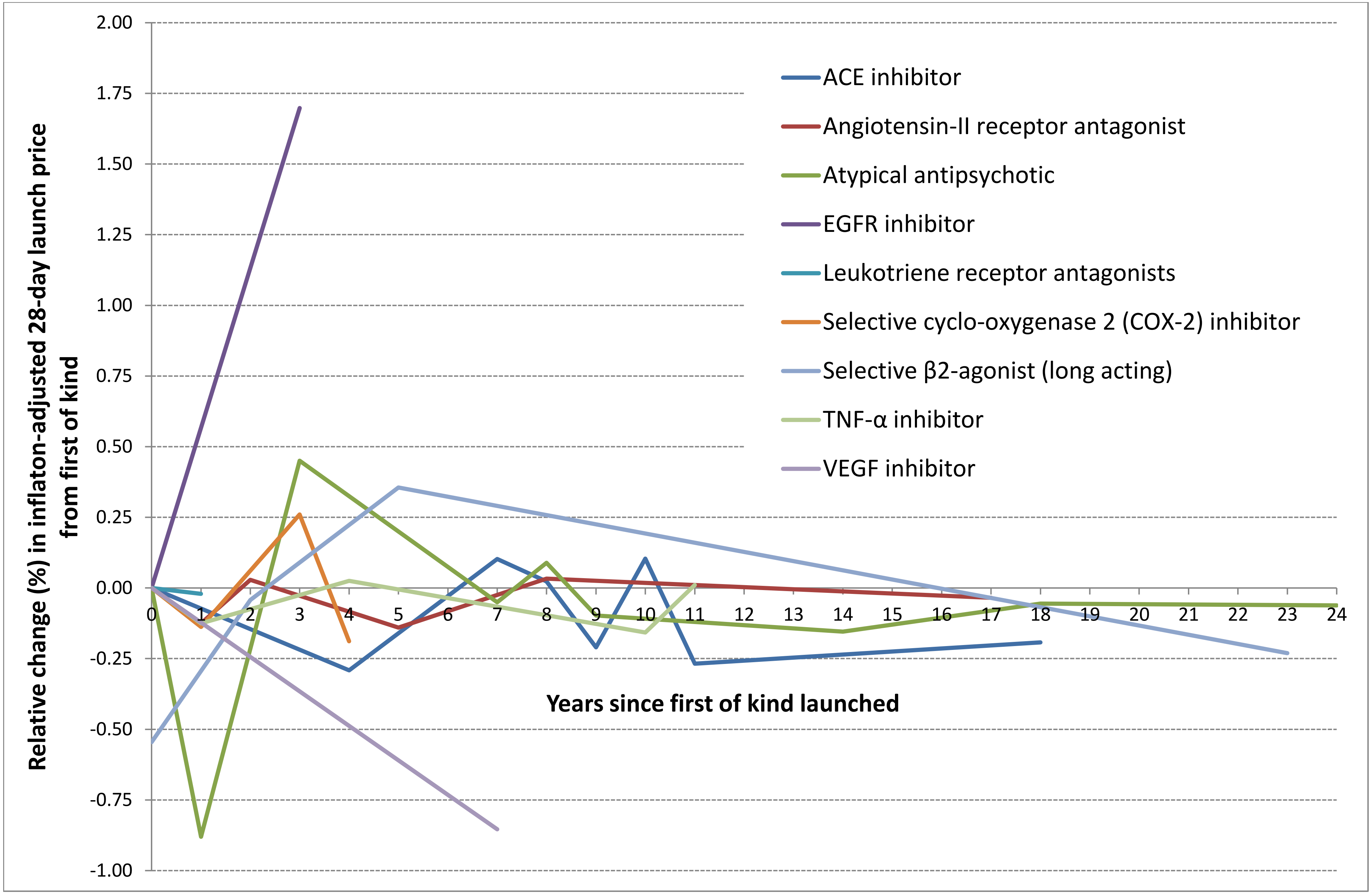
2.00
1.75
1.50
1.25
1.00
0.75
0.50
0.25
0.00
-0.25
-0.50
-0.75
-1.00
Relative change (%) in inflaton-adjusted 28-day launch price from first of kind
0 1 2 3 4 5 6 7 8 9 10 11 12 13 14 15 16 17 18 19 20 21 22 23 24
Years since first of kind launched
ACE inhibitor
Angiotensin-II receptor antagonist
Atypical antipsychotic
EGFR inhibitor
Leukotriene receptor antagonists
Selective cyclo-oxygenase 2 (COX-2) inhibitor
Selective β2-agonist (long acting)
TNF-α inhibitor
VEGF inhibitor

Disease area	Drug	Class/Mechanism	Year of launch	Adjusted 28-day launch price (£)	First of kind	Biologic drug	New drug or new indication
Hypertension	Captopril	ACE inhibitor	1981	59.06	Yes	No	New drug
Hypertension	Indoramin	α1-adrenergic receptor antagonist	1981	39.37	No	No	New drug
Hypertension	Dilitiazem	Calcium channel blocker	1984	49.82	No	No	New drug
Hypertension	Enalapril	ACE inhibitor	1985	41.85	No	No	New drug
Hypertension	Nicardipine	Calcium channel blocker	1986	45.52	No	No	New drug
Hypertension	Terazosin	α1-adrenergic receptor antagonist	1987	38.01	No	No	New drug
Hypertension	Bisoprolol fumarate	β-adrenergic receptor antagonist	1988	19.29	No	No	New drug
Hypertension	Lisinopril	ACE inhibitor	1988	47.91	No	No	New drug
Hypertension	Doxazosin	α1-adrenergic receptor antagonist	1989	32.02	No	No	New drug
Hypertension	Isradipine	Calcium channel blocker	1989	41.86	No	No	New drug
Hypertension	Quinapril	ACE inhibitor	1989	49.27	No	No	New drug
Hypertension	Amlodipine	Calcium channel blocker	1990	22.05	No	No	New drug
Hypertension	Perindopril	ACE inhibitor	1990	22.57	No	No	New drug
Hypertension	Ramipril	ACE inhibitor	1990	24.45	No	No	New drug
Hypertension	Celiprolol	β-adrenergic receptor antagonist	1991	17.13	No	No	New drug
Hypertension	Cilazapril	ACE inhibitor	1991	37.74	No	No	New drug
Hypertension	Felodipine	Calcium channel blocker	1991	14.20	No	No	New drug
Hypertension	Fosinopril	ACE inhibitor	1991	36.70	No	No	New drug
Hypertension	Esmolol	β-adrenergic receptor antagonist	1992	9.93	No	No	New drug
Hypertension	Lacidipine	Calcium channel blocker	1992	23.73	No	No	New drug
Hypertension	Trandolapril	ACE inhibitor	1992	20.86	No	No	New drug
Hypertension	Carvedilol	β-adrenergic receptor antagonist	1994	19.28	No	No	New drug
Hypertension	Torasemide	Loop diuretic	1994	7.41	No	No	New drug
Hypertension	Losartan	Angiotensin-II receptor antagonist	1995	27.26	Yes	No	New drug
Hypertension	Moxonidine	Imidazoline I_1 receptor agonist	1996	15.82	Yes	No	New drug
Hypertension	Valsartan	Angiotensin-II receptor antagonist	1996	23.85	No	No	New drug
Hypertension	Candesartan	Angiotensin-II receptor antagonist	1997	23.24	No	No	New drug
Hypertension	Irbesartan	Angiotensin-II receptor antagonist	1997	25.41	No	No	New drug
Hypertension	Lercanidipine	Calcium channel blocker	1998	14.77	No	No	New drug
Hypertension	Imidapril	ACE inhibitor	1999	9.51	No	No	New drug
Hypertension	Nebivolol	β-adrenergic receptor antagonist	1999	13.91	No	No	New drug
Hypertension	Eprosartan	Angiotensin-II receptor antagonist	2000	20.82	No	No	New drug
Hypertension	Telmisartan	Angiotensin-II receptor antagonist	2000	17.78	No	No	New drug
Hypertension	Olmesartan	Angiotensin-II receptor antagonist	2003	18.68	No	No	New drug
Hypertension	Aliskiren	Renin inhibitor	2007	23.71	Yes	No	New drug
Hypertension	Azilsartan medoxomil	Angiotensin-II receptor antagonist	2012	17.73	No	No	New drug
Asthma	Reproterol hydrochloride aerosol inhalation	Selective β2-agonist (short acting)	1981	9.22	No	No	New drug
Asthma	Reproterol hydrochloride tablets	Selective β2-agonist (short acting)	1981	14.20	No	No	New drug
Asthma	Hydrocortisone acetate	Corticosteroid	1982	8.64	No	No	New indication
Asthma	Budesonide	Corticosteroid	1983	14.59	No	No	New drug
Asthma	Pirbuterol aerosol inhalation	Selective β2-agonist (short acting)	1983	10.93	No	No	New drug
Asthma	Nedocromil sodium	Cromoglicate	1987	42.74	No	No	New drug
Asthma	Salmeterol	Selective β2-agonist (long acting)	1991	49.22	Yes	No	New drug
Asthma	Tulobuterol hydrochloride	Selective β2-agonist (long acting)	1991	22.37	No	No	New drug

For peer review only

Asthma	Bambuterol hydrochloride	Selective β2-agonist (long acting)	1993	20.27	No	No	New drug
Asthma	Pirbuterol tablets	Selective β2-agonist (short acting)	1993	12.13	No	No	New drug
Asthma	Fluticasone propionate	Corticosteroid	1994	17.53	No	No	New drug
Asthma	Formoterol fumarate	Selective β2-agonist (long acting)	1996	37.77	No	No	New drug
Asthma	Montelukast	Leukotriene receptor antagonists	1998	37.97	Yes	No	New drug
Asthma	Zafirlukast	Leukotriene receptor antagonists	1999	37.19	No	No	New drug
Asthma	Mometasone furoate	Corticosteroid	2003	29.74	No	No	New indication
Asthma	Ciclesonide	Corticosteroid	2006	9.67	No	No	New drug
Asthma	Omalizumab	Anti IgE humanised monoclonal antibody	2006	764.45	Yes	Yes	New drug
Asthma	Fluticasone furoate and vilanterol	Selective β2-agonist (long acting)	2014	26.43	No	No	New drug
Schizophrenia	Levomepromazine maleate	First generation antipsychotic	1983	20.22	No	No	New drug
Schizophrenia	Zuclopenthixol dihydrochloride	First generation antipsychotic	1983	19.41	No	No	New drug
Schizophrenia	Sulpiride #	Atypical antipsychotic	1984	139.22	No	No	New drug
Schizophrenia	Clozapine	Atypical antipsychotic	1990	331.80	Yes	No	New drug
Schizophrenia	Loxapine	First generation antipsychotic	1990	31.75	No	No	New drug
Schizophrenia	Remoxipride	Atypical antipsychotic	1991	39.75	No	No	New drug
Schizophrenia	Risperidone	Atypical antipsychotic	1993	189.15	No	No	New drug
Schizophrenia	Olanzapine	Atypical antipsychotic	1997	200.04	No	No	New drug
Schizophrenia	Sertindole	Atypical antipsychotic	1997	155.55	No	No	New drug
Schizophrenia	Amisulpride	Atypical antipsychotic	1998	93.95	No	No	New drug
Schizophrenia	Quetiapine	Atypical antipsychotic	1998	214.80	No	No	New drug
Schizophrenia	Zotepine	Atypical antipsychotic	1999	182.40	No	No	New drug
Schizophrenia	Aripiprazole	Atypical antipsychotic	2004	131.25	No	No	New drug
Schizophrenia	Paliperidone	Atypical antipsychotic	2008	112.92	No	No	New drug
Schizophrenia	Lurasidone	Atypical antipsychotic	2014	92.39	No	No	New drug
Rheumatoid arthritis	Tiaprofenic acid	Non-steroidal anti-inflammatory drug (NSAID)	1982	58.61	No	No	New drug
Rheumatoid arthritis	Etodolac	Non-steroidal anti-inflammatory drug (NSAID)	1985	71.92	No	No	New drug
Rheumatoid arthritis	Sulfasalazine	Aminosalicylate	1987	23.59	Yes	No	New indication
Rheumatoid arthritis	Nabumetone	Non-steroidal anti-inflammatory drug (NSAID)	1987	40.52	No	No	New drug
Rheumatoid arthritis	Tenoxicam	Non-steroidal anti-inflammatory drug (NSAID)	1988	40.38	No	No	New drug
Rheumatoid arthritis	Acemetacin	Non-steroidal anti-inflammatory drug (NSAID)	1991	28.64	No	No	New drug
Rheumatoid arthritis	Methotrexate	Dihydrofolate reductase inhibitor	1992	2.22	Yes	No	New indication
Rheumatoid arthritis	Ciclosporin	Calcineurin inhibitor	1994	240.63	Yes	No	New indication
Rheumatoid arthritis	Aceclofenac	Non-steroidal anti-inflammatory drug (NSAID)	1996	23.45	No	No	New drug
Rheumatoid arthritis	Meloxicam	Non-steroidal anti-inflammatory drug (NSAID)	1996	21.81	No	No	New drug
Rheumatoid arthritis	Leflunomide	Pyrimidine synthesis inhibitor	1999	69.71	Yes	No	New drug
Rheumatoid arthritis	Infliximab	TNF-α inhibitor	1999	1052.22	Yes	Yes	New indication
Rheumatoid arthritis	Rofecoxib	Selective cyclo-oxygenase 2 (COX-2) inhibitor	1999	30.12	Yes	No	New drug
Rheumatoid arthritis	Celecoxib	Selective cyclo-oxygenase 2 (COX-2) inhibitor	2000	25.97	No	No	New drug
Rheumatoid arthritis	Etanercept	TNF-α inhibitor	2000	920.27	No	Yes	New drug
Rheumatoid arthritis	Anakinra	IL-1 inhibitor	2002	888.47	Yes	Yes	New drug
Rheumatoid arthritis	Etoricoxib	Selective cyclo-oxygenase 2 (COX-2) inhibitor	2002	33.80	No	No	New drug
Rheumatoid arthritis	Adalimumab	TNF-α inhibitor	2003	946.83	No	Yes	New drug
Rheumatoid arthritis	Valdecoxib	Selective cyclo-oxygenase 2 (COX-2) inhibitor	2003	28.12	No	No	New drug
Rheumatoid arthritis	Rituximab	CD20 inhibitor	2006	330.65	Yes	Yes	New indication
Rheumatoid arthritis	Abatacept	Inhibitor of T-cell co-stimulation	2007	900.14	Yes	Yes	New drug

For peer review only

Rheumatoid arthritis	Tocilizumab	IL-6 inhibitor	2009	980.44	Yes	Yes	New drug
Rheumatoid arthritis	Certolizumab pegol	TNF-α inhibitor	2009	781.10	No	Yes	New drug
Rheumatoid arthritis	Golimumab	TNF-α inhibitor	2010	792.23	No	Yes	New drug
Colorectal cancer	Folinic acid + Fluorouracil (5FU)	Thymidylate synthase inhibitor	1992	83.04	No	No	New indication
Colorectal cancer	Raltitrexed	Thymidylate synthase inhibitor	1996	588.05	No	No	New drug
Colorectal cancer	Irinotecan	DNA topoisomerase I inhibitor	1997	1452.13	Yes	No	New drug
Colorectal cancer	Oxaliplatin	Platinum-based chemotherapeutic drug	2000	1195.80	Yes	No	New drug
Colorectal cancer	Capecitabine	Thymidylate synthase inhibitor	2001	507.49	No	No	New drug
Colorectal cancer	Tegafur (with uracil)	Thymidylate synthase inhibitor	2001	876.52	No	No	New drug
Colorectal cancer	Cetuximab	EGFR inhibitor	2005	1105.20	Yes	Yes	New drug
Colorectal cancer	Bevacizumab	VEGF inhibitor	2006	4200.67	Yes	Yes	New drug
Colorectal cancer	Panitumumab	EGFR inhibitor	2008	2981.95	No	Yes	New drug
Colorectal cancer	Regorafenib	Multi-tyrosine kinase inhibitor	2013	3888.39	Yes	No	New drug
Colorectal cancer	Aflibercept	VEGF inhibitor	2013	614.11	No	Yes	New drug

Clozapine is usually considered the first atypical antipsychotic to be launched, sulpiride was reclassified later.

For peer review only
